# Infrared Study of Electron-bombarded Phenanthrene (C_14_H_10_)/*Para*-H_2_ Matrices: Isomers of Protonated Phenanthrene (1‑, 3‑, 4‑, and 9‑H^+^C_14_H_10_)

**DOI:** 10.1021/acs.jpca.5c04756

**Published:** 2025-09-29

**Authors:** Jun-Ying Feng, Yuan-Pern Lee

**Affiliations:** † Department of Applied Chemistry and Institute of Molecular Science, 34914 National Yang Ming Chiao Tung University, Hsinchu 300093, Taiwan; ‡ Center for Emergent Functional Matter Science, 34914 National Yang Ming Chiao Tung University, Hsinchu 300093, Taiwan

## Abstract

Protonated polycyclic aromatic hydrocarbons are potential carriers of the unidentified infrared (UIR) emission bands observed in interstellar space. Laboratory-generated infrared (IR) spectra of these protonated species can aid in identifying the molecular origins of these bands. In this study, a mixture of phenanthrene (C_14_H_10_) and *p*-H_2_ was subjected to electron bombardment during deposition at 3.2 K, yielding new IR absorption features. These features decrease over time during maintenance of the matrix in darkness, consistent with the behavior of protonated species. Based on their photochemical responses to secondary laser irradiation at 619, 544, 524, and 463 nm, the features were categorized and assigned to four previously unreported isomers of protonated phenanthrene, 1-, 3-, 4-, and 9-H^+^C_14_H_10_. Spectral assignments were supported by comparisons with scaled harmonic vibrational wavenumbers and IR intensities of possible candidates predicted with the B3LYP/6–311++G­(d,p) method. The IR spectra of these isomers show prominent absorption in the 6–9 μm range but lack significant features near 11.3 μm, indicating that protonated phenanthrene is unlikely to be a major contributor to the UIR bands.

## Graphical abstract

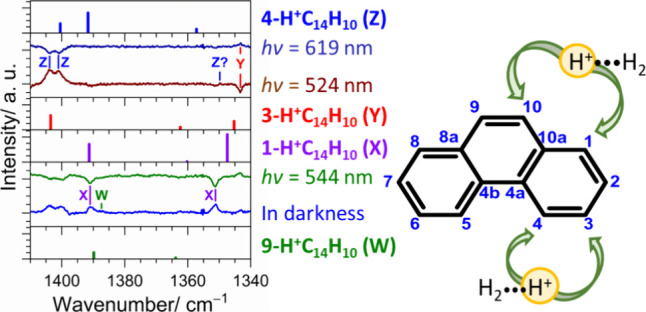

## 1 Introduction

The primary source of unidentified infrared (UIR) emission bands near 3.3, 6.2, 7.7, 8.6, 11.2, and 12.7 μm, observed in interstellar media, star-forming regions, and extragalactic objects, has been attributed to polycyclic aromatic hydrocarbons (PAH) and their derivatives, including cationic PAH (PAH^+^), protonated PAH (H^+^PAH), and hydrogenated PAH (HPAH).^1–3^ However, infrared (IR) spectra obtained from laboratory studies of PAH do not match closely the UIR bands. In outer space, abundant proton sources such as H_3_^+^ can readily transfer protons to PAH, which possess high proton affinity, forming protonated PAH (H^+^PAH). Similarly, H^+^PAH can also be produced by reactions between PAH^+^ and abundant H atoms. Due to their stable closed-shell structure, H^+^PAH exhibit low reactivity and can persist for extended periods in the interstellar environment.^4^ Beyond the potential role as UIR band carriers, theoretical investigations suggest that the electronic absorption spectra of H^+^PAH might also contribute to some diffuse interstellar bands (DIB) in the visible region.^4,5^

Large PAH and their derivatives are considered as potential carriers of UIR bands, as previous research suggests that PAH with fewer than 50 carbon atoms are difficult to preserve in space for a long time due to the intense UV field in space.^6^ However, small PAH serve as essential building blocks for larger PAH with higher carbon content, making their study particularly important. The bottom-up synthesis of larger PAH from smaller aromatic species is feasible in the gaseous phase via radical–radical reactions and ion–molecule reactions.^7,8^ In particular, the reaction of naphthalene (C_1__0_H_8_) and fulvenallene (C_7_H_6_) leading to the formation of phenanthrene (C_1__4_H_1__0_) has been experimentally demonstrated.^9,10^ The smallest PAH, naphthalene, consists of two fused benzene rings; the infrared spectra of its protonated isomers (1-H^+^C_10_H_8_ and 2-H^+^C_10_H_8_) have been reported.^11–15^

Phenanthrene (C_14_H_10_), an isomer of anthracene, consists of three fused benzene rings. In this paper, the formula C_14_H_10_ is used to represent only phenanthrene, not anthracene. Cané et al. recorded the IR absorption spectra of gaseous C_14_H_10_ and deuterated phenanthrene (C_14_D_10_).^16^ Hudgins et al. reported the IR absorption spectra of C_14_H_10_ in an Ar matrix; the observed vibrational wavenumbers agree satisfactorily with the scaled harmonic vibrational wavenumbers predicted with the B3LYP/6–31G* method.^17,18^ C_14_H_10_ has seven sites for protonation or hydrogenation at positions 10, 10a, 1, 2, 3, 4, and 4a, which are equivalent to sites 9, 8a, 8, 7, 6, 5, and 4b due to symmetry considerations, as shown in Figure 1. Following previous literatures, positions 9 and 8a are preferred designations instead of 10 and 10a. Saed et al. computed the relative energies of various isomers of protonated C_14_H_10_ (H^+^C_14_H_10_) at the MP2/cc-pVTZ level of theory, identifying 9-H^+^C_14_H_10_ as the least-energy isomer.^19^ Garkusha et al. generated H^+^C_14_H_10_ from 9,10-dihydro-phenanthrene using a hot-cathode discharge source. After matrix deposition of the ions in Ne at 6 K, the recorded electronic absorption spectra revealed progressions assignable to 1-, 2-, 3-, 4-, and 9-H^+^C_14_H_10_, with no evidence of protonation on the fused ring at positions 4a and 8a.^20^ Knorke et al. reported the infrared multiphoton dissociation (IRMPD) spectrum of protonated anthracene, but no assignment on the protonation site was provided.^21^ To date, IR spectra of H^+^C_14_H_10_ remain unreported.

**Figure 1:**
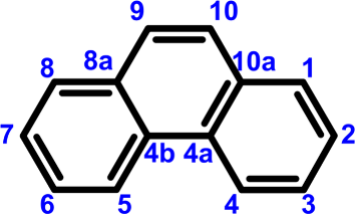
Carbon numbers of phenanthrene. Because of the symmetry, numbers 9, 8a, 8, 7, 6, 5, and 4b are equivalent to 10, 10a, 1, 2, 3, 4, and 4a, respectively.

In this study, we employed electron-bombardment on a mixture of C_14_H_10_ and *p*-H_2_ during matrix deposition at 3.2 K and recorded IR spectra of four low-lying protonated isomers, 1-, 3-, 4-, and 9-H^+^C_14_H_10_, in solid *p*-H_2_.

## 2 Methods

The IR absorption spectroscopy of species isolated in solid *p*-H_2_ has been previously described.^22–24^ A Ni-plated oxygen-free high-conductivity copper (OFHC) plate, mounted on a closed-cycle He refrigerator (Sumitomo CSW-71), was cooled to 3.2 K and served as both a matrix substrate and a reflector for double-path absorption. IR absorption spectra were acquired using a Fourier-transform infrared (FTIR) spectrometer (Bruker, VERTEX 80 V) equipped with a KBr beam splitter and a mercury–cadmium–telluride (HgCdTe) detector at 77 K. IR spectra covering a spectral range of 500–3200 cm^–1^ were recorded at a resolution of 0.25 cm^–1^; typically, 600 scans were averaged at each stage of the experiment.

The conversion of *normal*-H_2_ (*o*-H_2_: *p*-H_2_ = 3:1) to *p*-H_2_ has been reported previously.^25,26^ *Normal*-H_2_ was passed through a hydrated iron­(III) oxide catalyst (Sigma-Aldrich, catalyst grade, 30–50 mesh) maintained at 13.3 K. The mole fraction of *o*-H_2_ was estimated to be ∼ 1200 ppm (part per million) following the method reported by Tam and Fajardo.^27^

Gaseous mixtures of C_14_H_10_/*p*-H_2_ were prepared by passing *p*-H_2_ over solid C_14_H_10_ at 298 K. The resulting mixture, flowing at a rate of ∼ 19 mmol h^–1^, was deposited onto the cold substrate during electron bombardment for 6 h. The electron gun was operated at an energy of 300 eV and a current of 20 μA. Following deposition, the matrix was maintained in darkness for 16 h to facilitate the neutralization of H^+^C_14_H_10_ by electrons trapped in the matrix. During this process, H^+^C_14_H_10_ decreased while HC_14_H_10_ increased. Other electron-bombarded C_14_H_10_/*p*-H_2_ matrices were prepared and subsequently irradiated at 619, 544, 524, and 463 nm (10–20 mW at 10 Hz) using an OPO laser (EKSPLA, NT340) to categorize spectral features based on their photolytic behavior. Phenanthrene (C_14_H_10_, Chem Service, 98.7%) was used as received. The IR studies of HC_14_H_10_ will be discussed in the forthcoming paper.

Quantum-chemical calculations were performed using the Gaussian 16 program.^28^ Geometrical optimizations and vibrational analyses were carried out using the B3LYP hybrid functionals^29,30^ and the standard basis set 6–311++G­(d,p).^31^ Harmonic vibrational wavenumbers for all species considered in this work were scaled according to the equation *y* = (0.9548 ± 0.0035) *x* + (27.9 ± 7.2) cm^–1^, in which *y* represents the scaled vibrational wavenumber and *x* denotes the computed harmonic vibrational wavenumber. This scaling equation was derived from a linear fit of observed wavenumbers of C_14_H_10_ in *p*-H_2_ versus calculated harmonic vibrational wavenumbers in the range 500–3200 cm^–1^. To improve accuracy below 2000 cm^–1^, an alternative scaling equation *y* = (0.9804 ± 0.0027) *x* + (2.33 ± 2.88) cm^–1^ was used, based on fitted data within the spectral range 500–2000 cm^–1^. The first equation was applied only for wavenumbers above 2000 cm^–1^. Anharmonic vibrational wavenumbers and IR intensities were calculated by employing a second-order vibrational perturbation theory (VPT2) implemented in Gaussian 16.^32^ Single-point ground-state electronic energies were computed using the coupled-cluster method with single, double, and perturbative triple excitations, CCSD­(T),^33^ based on geometries optimized with the B3LYP/6–311++G­(d,p) method. Zero-point vibrational energies (ZPVE) were corrected according to harmonic vibrational wavenumbers calculated with the B3LYP/6–311++G­(d,p) method.

## 3 Results

### 3.1 Computational Results

The geometries of seven possible isomers of protonated phenanthrene (H^+^C_14_H_10_), optimized with the B3LYP/6–311++G­(d,p) method, are presented in Figure S1. Their energies were calculated with the CCSD­(T)/6–311++G­(d,p)//B3LYP/6–311++G­(d,p) method and corrected for zero-point vibrational energy using the B3LYP/6–311++G­(d,p) method. The carbon skeletons of 4a- and 8a-H^+^C_14_H_10_ are nonplanar, whereas those of other isomers remain planar.

Table 1 provides the relative energies of all isomers of H^+^C_14_H_10_ with respect to 9-H^+^C_14_H_10_, along with the barriers for proton transfer to adjacent sites for each isomer, as illustrated in Figure S2. The exothermicity of 407 kJ mol^–1^ for the proton-transfer reaction H_3_^+^ + C_14_H_10_ → H_2_ + 9-H^+^C_14_H_10_ implies a proton affinity of 829 kJ mol^–1^ for C_14_H_10_ at site 9. Isomers 1-, 3-, and 4-H^+^C_14_H_10_ exhibit relative energies within 4 kJ mol^–1^ of 9-H^+^C_14_H_10_, whereas 2-H^+^C_14_H_10_ is slightly higher at 8.4 kJ mol^–1^. In contrast, the energies of 4a- and 8a-H^+^C_14_H_10_ exceed that of 9-H^+^C_14_H_10_ by 48 and 58 kJ mol^–1^, respectively. The barriers for proton transfer to adjacent carbon sites are generally in the range of 60–70 kJ mol^–1^, except for transfers from site 10a to sites 1 and 10 and from site 4a to sites 10a and 4.

**Table 1:** Relative Energies (kJ mol^–1^) of Isomers of H^+^C_14_H_10_ and Transition States (TS) for Proton Transfer Calculated with the CCSD­(T)/6-311++G­(d,p)//B3LYP/6–311++G­(d,p) Method

		H^+^ transfer		
species	rel. energy^a^	transfer	TS energy^b^	barrier
	/kJ mol^–1^		/kJ mol^–1^	/kJ mol^–1^
9-H^+^C_14_H_10_ + H_2_	0.0	10 (9)^c^ → 10a	98.2	98.2
8a-H^+^C_14_H_10_ + H_2_	58.0	10a (8a)^d^ → 1	86.2	28.2
1-H^+^C_14_H_10_ + H_2_	2.5	1 → 2	64.5	62.0
2-H^+^C_14_H_10_ + H_2_	8.4	2 → 3	78.2	69.8
3-H^+^C_14_H_10_ + H_2_	3.9	3 → 4	64.1	60.2
4-H^+^C_14_H_10_ + H_2_	3.6	4 → 4a	83.1	79.5
4a-H^+^C_14_H_10_ + H_2_	47.7	4a → 10a	85.3	37.6
C_14_H_10_ + H_3_^+^	406.9			

a Zero-point vibrational energies (ZPVE) were corrected according to harmonic vibrational wavenumbers calculated with the B3LYP/6–311++G­(d,p) method.

b Transition structure (TS) energy for H^+^ transfer is relative to the energy of 10-H^+^C_14_H_10_ + H_2_.

c Sites 9 and 10 are equivalent. Conventionally, the preferred designation is 9-H^+^C_14_H_10_.

d Sites 8a and 10a are equivalent. Conventionally, the preferred designation is 8a-H^+^C_14_H_10_

The scaled harmonic vibrational wavenumbers and IR intensities of 9-, 1-, 2-, 3-, 4-, 4a-, and 8a-H^+^C_14_H_10_ are presented in Tables S1–S3; their IR stick spectra are compared with that of C_14_H_10_ in Figure S3. The anharmonic vibrational wavenumbers and IR intensities of 9-, 1-, 2-, 3-, and 4-H^+^C_14_H_10_ are also presented in Tables S1 and S2. Protonation activates vibrational modes in region 1100–1650 cm^–1^, but diminishes intense modes of C_14_H_10_ in regions 700–850 and 3000–3100 cm^–1^. The characteristic CH_2_-stretching modes of 9-, 1-, 2-, 3-, and 4-H^+^C_14_H_10_ appear near 2850 cm^–1^. Anharmonic calculations predicted many combination bands in the region 1350–1650 cm^–1^ with significant intensity. However, our experimental data show no clear evidence of these combination bands.

### 3.2 IR spectrum of C_14_H_10_/*p*-H_2_

Figure S4 compares the infrared spectrum of a C_14_H_10_/*p*-H_2_ matrix (trace a) in regions 600–1970 and 2950–3150 cm^–1^ with the previously reported spectrum of C_14_H_10_ in an Ar matrix (trace b).^17^ The IR spectrum of C_14_H_10_ in solid *p*-H_2_ is characterized by two intense bands at 736.2 and 813.5 cm^–1^, along with several weaker features in regions 800–1550 and 3040–3100 cm^–1^. The observed band positions are comparable to those found in solid Ar and in the gaseous phase.^16^ These experimental spectra are further compared with the stick spectrum simulated according to scaled harmonic vibrational wavenumbers and IR intensities predicted for C_14_H_10_ using the B3LYP/6–311++G­(d,p) method (trace c), demonstrating satisfactory agreement except in the CH-stretching region. Table S4 provides a detailed comparison between the observed wavenumbers and relative IR intensities of C_14_H_10_ in solid *p*-H_2_, solid Ar, and the gaseous state, alongside the scaled harmonic vibrational wavenumbers and predicted IR intensities. The mean absolute deviation between experimental results and the scaled harmonic vibrational wavenumbers in this study is 3.3 ± 4.0 cm^–1^, while that between the gaseous experiments and the scaled harmonic vibrational wavenumbers is 5.1 ± 6.5 cm^–1^. The mean absolute deviation between spectral data obtained in solid Ar and solid *p-*H_2_ is 1.8 ± 2.1 cm^–1^, while that between values in the gaseous phase and in solid *p-*H_2_ is 2.4 ± 3.0 cm^–1^.

### 3.3 IR Spectra of Protonated Phenanthrene

Figure 2 presents the representative IR spectra of an electron-bombarded C_14_H_10_/*p*-H_2_ matrix in region 1328–1480 cm^–1^, recorded after various experimental steps. Spectra covering an extended region of 575–1640 cm^–1^ are shown in Figure S5. For comparison, the spectrum of C_14_H_10_/*p*-H_2_ without electron bombardment is provided in Figures 2a and S5a. After 6 h of deposition with electron bombardment, several new spectral features emerged, as shown in Figures 2b and S5b. Subsequently, the matrix was maintained in darkness for 16 h to allow most protonated species to neutralize via combination with remnant electrons trapped in the *p*-H_2_ matrix; the difference spectrum after this step is presented in Figures 2c and S5c. Some absorption features decreased by 26–59%, suggesting that they are likely due to H^+^C_14_H_10_. The mixing ratios of each species after deposition and prolonged maintenance in darkness are summarized in Table 2; the percentage variations are listed in parentheses. To categorize spectral features that diminished after prolonged darkness, a second electron-bombarded matrix was prepared under similar conditions. Sequential secondary irradiations at 619, 544, 524, and 463 nm (each using laser energies ∼ 1.5 mJ at 10 Hz for 20 min) were consecutively performed. The resulting difference spectra, obtained on subtracting the spectrum recorded before irradiation at a specific wavelength from that recorded after such irradiation, are shown in Figures 2d–2g and S5d–5g, respectively.

**Figure 2:**
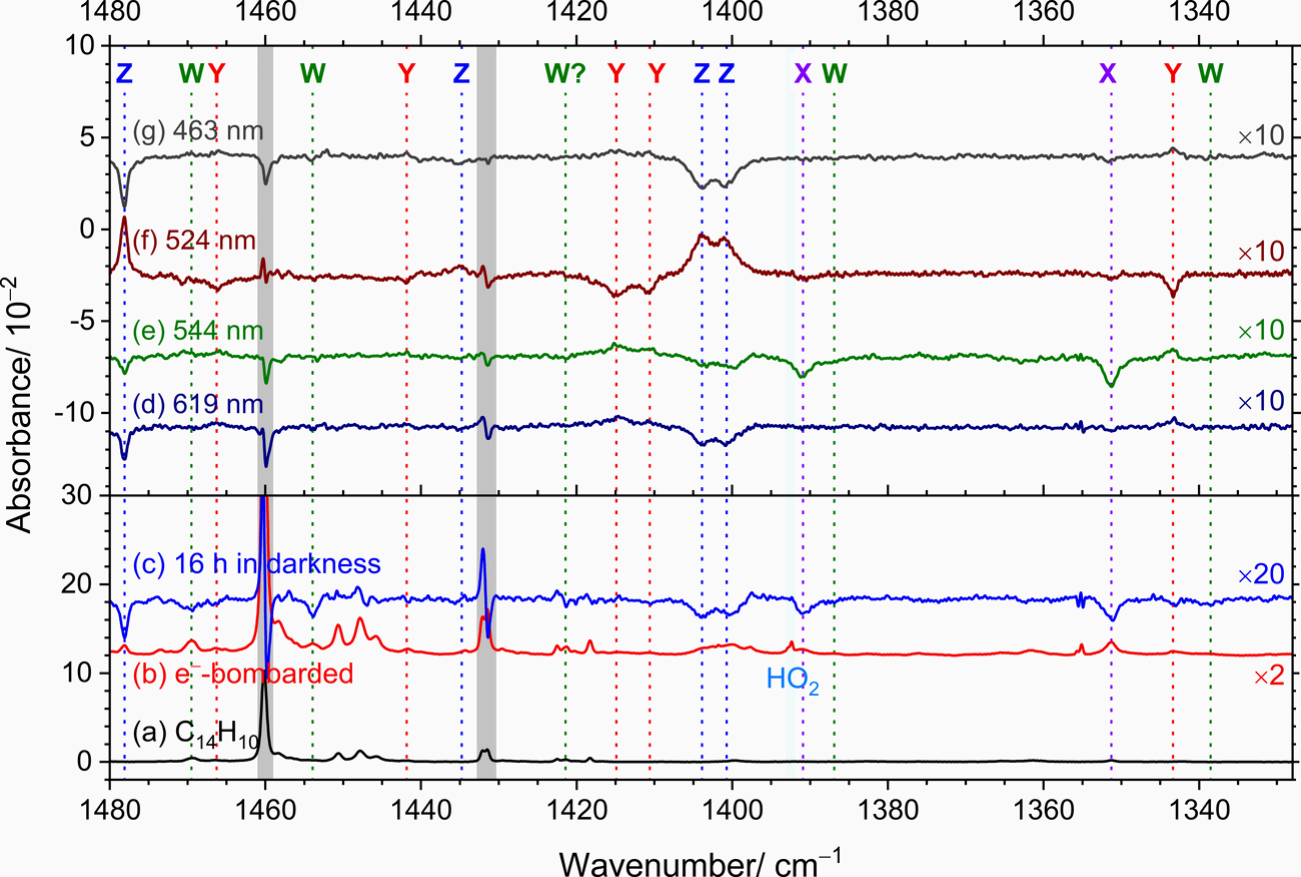
Representative infrared spectra of an electron-bombarded C_14_H_10_/*p*-H_2_ matrix after each experimental step. (a) C_14_H_10_/*p*-H_2_ matrix without electron bombardment. (b) Electron-bombarded C_14_H_10_/*p*-H_2_ matrix. (c) Difference spectrum measured after maintenance of the matrix in darkness for 16 h in a separate experiment. Difference spectra after secondary irradiation at 619 (d), 544 (e), 524 (f), and 463 nm (g); each irradiation step is 20 min. Bands in groups W, X, Y, and Z are indicated with color-coded labels and dashed lines. Spectral regions subjected to interference from the intense absorption of C_14_H_10_ are shaded gray. Baselines were shifted for clarity.

**Table 2:** Summary of Estimated Mixing Ratios of Isomers of H^+^C_14_H_10_ after Each Experimental Step

	experiment A					experiment B	
	deposition^a^	619 nm	544 nm	524 nm	463 nm	deposition^a^	16 h in darkness
assignment (group)	ppb^b^	ppb^b^	ppb^b^	ppb^b^	ppb^b^	ppb^b^	ppb^b^
9-H^+^C_14_H_10_ (W)	132 ± 27	132 ± 27	132 ± 27	132 ± 27	119 ± 10	110 ± 56	68 ± 21
	(100%)	(0%)	(0%)	(0%)	(−10%)	(100%)	(−38%)
1-H^+^C_14_H_10_ (X)	78 ± 14	78 ± 14	29 ± 17	13 ± 10	9 ± 10	121 ± 11	90 ± 5
	(100%)	(0%)	(−63%)	(−55%)	(−31%)	(100%)	(−26%)
3-H^+^C_14_H_10_ (Y)	128 ± 42	173 ± 27	235 ± 40	97 ± 73	142 ± 19	140 ± 77	58 ± 10
	(100%)	(+35%)	(+36%)	(−59%)	(+46%)	(100%)	(−59%)
4-H^+^C_14_H_10_ (Z)	120 ± 37	64 ± 36	∼0	119 ± 41	65 ± 56	147 ± 50	75 ± 16
	(100%)	(−47%)	(−100%)	appeared	(−45%)	(100%)	(−49%)

a Mixing ratios after depositions with electron bombardment.

b The percentage variation of absorbance in each step is written in parentheses.

These features can be classified into four groups, designated as groups W to Z in Figures 2 and S5, according to their distinct behavior upon secondary irradiation at various wavelengths. The mixing ratios of each species after each experimental step are summarized in Table 2; the percentage variations observed after each irradiation step are listed in parentheses. As shown in Figures 2 and S5, features in group X, indicated with violet dashed lines and labeled X, remained nearly unchanged at 619 nm, decreased by ∼ 49 ppb (63%), 16 ppb (55%), and 4 ppb (31%) at 544, 524, and 463 nm, respectively; the numbers in parentheses indicate percentage loss during a specific irradiation step. Features in group Y increased by ∼ 45 ppb (35%) and 62 ppb (36%) on irradiation at 619 and 544 nm, respectively, decreased by ∼ 138 ppb (59%) on irradiation at 524 nm, and increased again by ∼ 45 ppb (46%) on irradiation at 463 nm; they are indicated by red dashed lines and labeled Y. In contrast to group Y, features in group Z decreased by ∼ 56 ppb (47%) on irradiation at 619 nm and became nearly completely vanished after irradiation at 544 nm; they recovered (∼119 ppb) on irradiation at 524 nm and decreased by ∼ 54 ppb (45%) on irradiation at 463 nm. These features are indicated by blue dashed lines and labeled Z. Features in group W remained nearly the same after irradiation at 619, 544, and 524 nm (no change in difference spectra) and decreased slightly by ∼ 13 ppb (10%) upon irradiation at 463 nm; these features are indicated with green dashed lines and labeled W.

To identify features in the CH-stretching region, presented in Figure S6, is challenging, as C_14_H_10_ has intense CH-stretching bands but isomers of H^+^C_14_H_10_ were predicted to have weak CH-stretching bands (Figure S3). For the characteristic CH_2_-stretching region, two broad features decreased in darkness. We could identify one feature near 2840.9 cm^–1^ in group Y that decreased significantly upon irradiation at 524 nm and increased at 463 nm, and another one near 2848.8 cm^–1^ in group X that decreased significantly at 544 nm. As the band maximum near 2840.9 cm^–1^ observed after maintenance in darkness has a wavenumber larger than that of the band in group X, we tentatively assigned it to group W. As discussed in Sections 4.1–4.2, these features in groups W–Z are assigned to 9-, 1-, 3-, and 4-H^+^C_14_H_10_, respectively,

## 4 Discussion

### 4.1 Assignments of Features in Group W to 9-H^+^C_14_H_10_ and Group X to 1-H^+^C_14_H_10_

Representative spectral features in groups W and X, observed in region 1340–1490 cm^–1^, are compared with stick spectra of five low-energy isomers of H^+^C_14_H_10_ in Figure 3. These simulations were based on scaled harmonic vibrational wavenumbers and IR intensities predicted with the B3LYP/6–311++G­(d,p) method. A comparison of experimental features of groups W and X in 575–1640 cm^–1^ with predicted stick spectra of all seven isomers of H^+^C_14_H_10_ is presented in Figures S7 and S8, respectively. The inverted difference spectra, measured after maintaining the matrix in darkness, are shown in Figures 3b, S7a, and S8a. The difference spectra after irradiation at 544 nm, presented in Figures 3c and S8b, illustrate the most pronounced changes in group X. In addition, the difference spectra after irradiation at 463 nm are presented in Figure S7b because features in group W decreased slightly only at this wavelength. Features of groups W and X are indicated with green and violet lines and labels, respectively. The predicted stick spectra based on the anharmonic calculations of 9- and 1-H^+^C_14_H_10_ are presented in Figures S7d and S8e, respectively.

**Figure 3:**
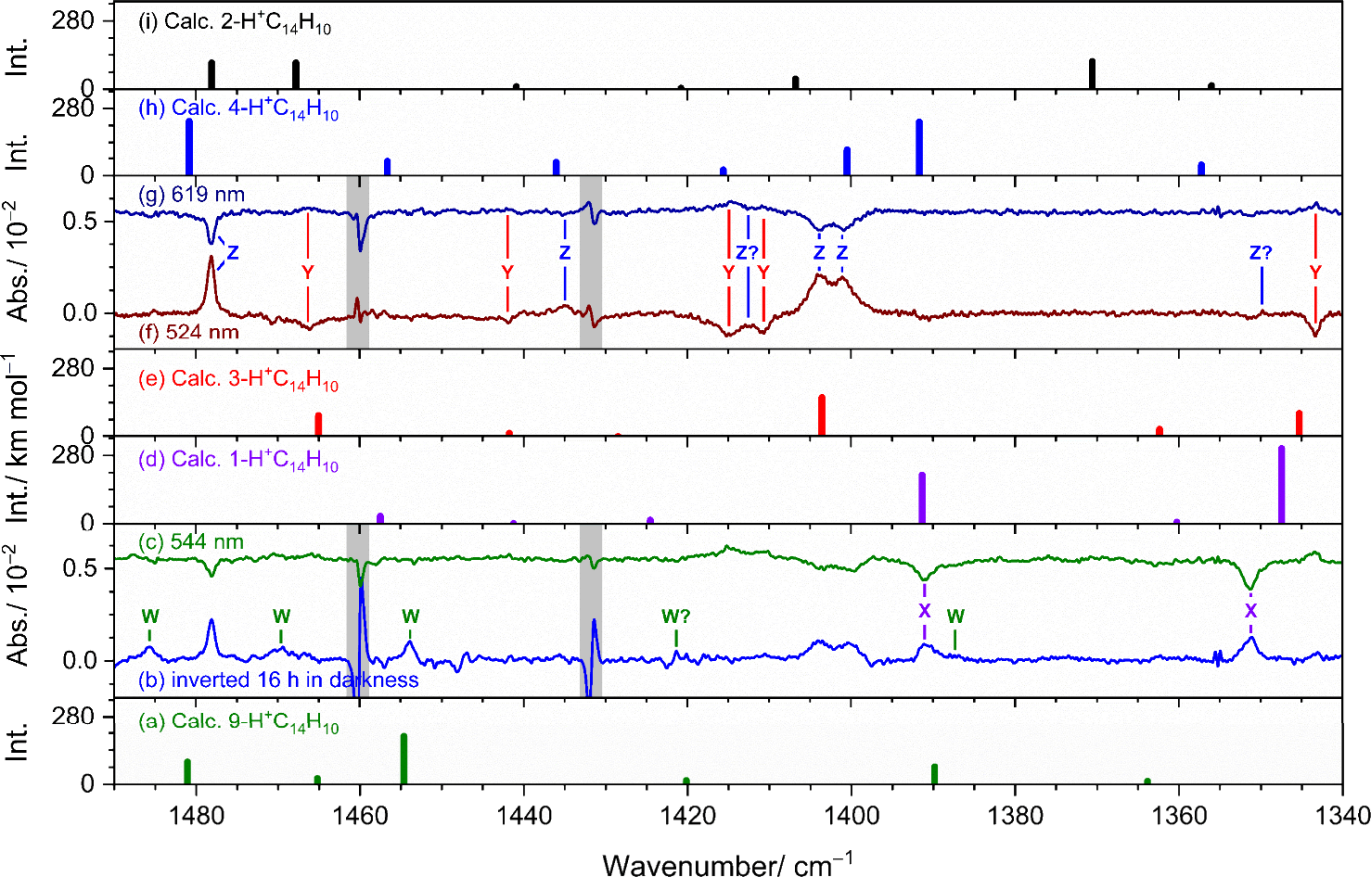
Comparison of bands in groups W, X, Y, and Z in region 1340–1490 cm^–1^ with stick spectra of 9-H^+^C_14_H_10_ (a), 1-H^+^C_14_H_10_ (d), 3-H^+^C_14_H_10_ (e), 4-H^+^C_14_H_10_ (h), and 2-H^+^C_14_H_10_ (i) according to scaled harmonic vibrational wavenumbers and IR intensities calculated with the B3LYP/6–311++G­(d,p) method. Panels (b), (c), (f), and (g) are experimental spectra taken from Figure 2 . (b) Inverted difference spectrum measured after maintenance of the matrix in darkness for 16 h. Difference spectra after secondary irradiation at 544 (c), 524 (f), and 619 nm (g). Bands in groups W, X, Y, and Z are indicated with color-coded labels and solid lines. Spectral regions subjected to interference from the intense absorption of C_14_H_10_ are shaded gray. Baselines were shifted for clarity.

The observed wavenumbers and relative intensities of bands in group W show the closest agreement with those predicted for 9-H^+^C_14_H_10_ (Figures 3a and S7c, in green) but align less well with other isomers of H^+^C_14_H_10_ (Figure S7). The two most intense features, recorded at 1611.5 and 1453.9 cm^–1^, correspond closely to the scaled harmonic vibrational wavenumber predicted near 1623 and 1455 cm^–1^ (both associated with C=C stretching modes) for 9-H^+^C_14_H_10_. Additionally, six medium-intensity features were observed at 1485.7, 1387.5, 1339.0, 1257.8, 1178.9, and 772.4 cm^–1^, showing good agreement with those predicted near 1481, 1390 (both C=C stretching modes), 1334 (ring deformation coupled with CH_2_-scissoring mode), 1258, 1182 (both in-plane CH-bending modes), and 774 cm^–1^ (out-of-plane CH-bending mode). Furthermore, six additional weaker features also match with theoretical predictions, as summarized in Table 3, bringing the total number of identified vibrational modes to 14. All vibrational modes predicted with IR intensity >20 km mol^–1^ are listed in Table 3. Most vibrational modes were observed, except for five predicted near 1612, 1579, 1533, 1309, and 718 cm^–1^, which were obscured by absorption of features in group Y or C_14_H_10_. The characteristic CH_2_-stretching (ν_10_) mode predicted near 2864 cm^–1^ with an IR intensity close to 31 km mol^–1^ is tentatively assigned to a feature at 2852.4 cm^–1^, which decreased in darkness but remained nearly unchanged after photolysis; this band overlaps with a band near 2849 cm^–1^ of group X. Thus, the 14 features in group W are assigned to 9-H^+^C_14_H_10_. The mean absolute deviation between observed wavenumbers and scaled harmonic vibrational wavenumbers of 9-H^+^C_14_H_10_ is calculated as 4.1 ± 3.7 cm^–1^. The largest deviation is 11.9 cm^–1^ for ν_10_, which is typical for the CH_2_-stretching mode of protonated PAH. The anharmonic vibrational calculations yield a value of 2826 cm^–1^, suggesting that the true value might be smaller than the scaled harmonic vibrational wavenumbers.

**Table 3:** Comparison of Experimental Vibrational Wavenumbers and Relative IR Intensities of 9- and 1-H^+^C_14_H_10_ with Those Predicted with the B3YLP/6–311++G­(d,p) Method

9-H^+^C_14_H_10_ (group W)						1-H^+^C_14_H_10_ (group X)					
mode	sym.	calculation		*p*-H_2_		mode	sym.	calculation		*p*-H_2_	
*v*_10_	a′	2864.3^a^	(31)^b^	2852.4?	(−)^c^	*v*_10_	a′	2873.0^a^	(31)^b^	2848.8	(−)^d^
*v*_11_	a′	1623.1	(222)	1611.5	(100)^e^	*v*_11_	a′	1616.4	(50)	1610.4	(41)^e^
*v*_12_	a′	1611.8	(55)	^f^		*v*_13_	a′	1575.0	(135)	1579.5	(44)
*v*_13_	a′	1579.0	(25)	^f^		*v*_14_	a′	1546.7	(29)		^g^
*v*_15_	a′	1532.5	(39)	^g^		*v*_15_	a′	1530.8	(201)	1523.4	(42)
*v*_16_	a′	1481.1	(91)	1485.7	(41)	*v*_16_	a′	1507.9	(52)		^g^
*v*_17_	a′	1465.2	(24)	1470.0	(25)	*v*_17_	a′	1457.5	(31)		^g^
*v*_18_	a′	1454.6	(197)	1453.9	(91)	*v*_20_	a′	1391.3	(200)	1390.8	(84)
*v*_19_	a′	1420.2	(16)	1421.2?	(26)	*v*_22_	a′	1347.4	(309)	1351.1	(100)
*v*_20_	a′	1389.8	(72)	1387.5	(35)	*v*_23_	a′	1327.3	(222)	1296.0	(61)
*v*_22_	a′	1333.5	(89)	1339.0	(51)	*v*_26_	a′	1251.7	(81)	1250.2	(29)
*v*_23_	a′	1326.5	(112)	1325.2	(26)	*v*_27_	a′	1234.0	(21)		^h^
*v*_24_	a′	1308.9	(72)	^g^		*v*_31_	a′	1158.8	(24)	1156.7	(13)
*v*_26_	a′	1257.5	(70)	1257.8	(32)	*v*_56_	a″	830.3	(40)	827.6	(12)
*v*_29_	a′	1182.2	(45)	1178.9	(53)	*v*_57_	a″	762.3	(47)	763.4	(39)
*v*_30_	a′	1177.8	(21)	1172.0	(24)	*v*_59_	a″	719.8	(25)		^h^
*v*_31_	a′	1150.0	(20)	1147.9	(10)						
*v*_57_	a″	774.1	(76)	772.4	(29)						
*v*_59_	a″	717.6	(23)	^h^							

a Harmonic vibrational wavenumbers (cm^–1^) are scaled according to 0.9548*x* + 27.9 for wavenumbers >2000 cm^–1^ and 0.9804*x* + 2.33 for wavenumbers <2000 cm^–1^.

b Harmonic IR intensities (in km mol^–1^) are listed in parentheses. Only those with intensities >20 km mol^–1^ are listed.

c Interfered by group X.

d Interfered by group W.

e Percentage integrated intensities of each isomer relative to the most intense band are listed in parentheses.

f Interfered by group Y.

g Interfered by C_14_H_10_.

h Too weak to be observed.

The spectral pattern (wavenumbers and relative IR intensities) of group X agrees satisfactorily with scaled harmonic vibrational predictions for 1-H^+^C_14_H_10_ (Figures 3d and S8d, in violet), but shows less agreement with other isomers of H^+^C_14_H_10_ (Figure S8). The two most intense features, observed at 1351.1 and 1390.8 cm^–1^, correspond closely to scaled harmonic vibrational wavenumber predicted at 1347 and 1391 cm^–1^ (both C=C stretching modes) for 1-H^+^C_14_H_10_. Additionally, five medium-intensity features were recorded at 1579.5, 1523.4, 1296.0, 1250.2, and 763.4 cm^–1^, showing good agreement with those predicted near 1575, 1531 (both C=C stretching modes), 1327 (CH_2_ scissoring mode), 1252 (in-plane CH-bending mode), and 762 cm^–1^ (out-of-plane CH-bending mode). For the characteristic symmetric CH_2_-stretching mode, a band at 2848.8 cm^–1^ is assigned to group X (Figure S6), as its decrease is significant upon irradiation at 544 nm; its wavenumber is near the scaled harmonic vibrational wavenumber predicted at 2873 cm^–1^. As summarized in Table 3, which lists all vibrational modes with IR intensity >20 km mol^–1^, a total of 11 bands were identified for 1-H^+^C_14_H_10_. All vibrational modes predicted with IR intensity greater than 30 km mol^–1^ were observed, except for two predicted near 1508 and 1458 cm^–1^, which were interfered by C_14_H_10_ absorption. Thus, the bands in group X are assigned to 1-H^+^C_14_H_10_. The mean absolute deviation between observed wavenumbers and scaled harmonic vibrational wavenumbers for 1-H^+^C_14_H_10_ is determined to be 7.7 ± 10.2 cm^–1^. The largest deviation of 31 cm^–1^ is for ν_23_, a band predicted near 1327 cm^–1^ for the CH_2_ scissoring mode but observed at 1296.0 cm^–1^. However, the anharmonic vibrational calculations predicted it to be 1307 cm^–1^ (Table S1 and Figure S8), only 11 cm^–1^ larger than the observed value.

### 4.2 Assignment of Features in Groups Y to 3-H^+^C_14_H_10_ and Z to 4-H^+^C_14_H_10_

The experimental spectra of features in groups Y and Z in region 1340–1490 cm^–1^ are compared in Figure 3 with stick spectra of five low-lying isomers of H^+^C_14_H_10_, simulated based on scaled harmonic vibrational wavenumbers and IR intensities predicted with the B3LYP/6–311++G­(d,p) method. Bands in groups Y and Z are marked with red and blue labels and lines, respectively. The difference spectrum obtained after secondary irradiation at 619 nm (Figure 3g) is presented, as only features in groups Y and Z showed significant variation after irradiation at this wavelength, with the former increased by ∼ 35%, while the latter decreased by ∼ 47%. Following irradiation at 524 nm, features in group Z showed a substantial increase, whereas those in group Y decreased by ∼ 59%, as depicted in Figure 3f. A more comprehensive comparison of spectral features in groups Y and Z, in region 575–1640 cm^–1^, with all seven isomers of H^+^C_14_H_10_ is provided in Figures S9 and S10, respectively. The predicted stick spectra based on the anharmonic calculations of 3- and 4-H^+^C_14_H_10_ are presented in Figures S9g and S10h, respectively.

The observed wavenumbers of bands in group Y agree satisfactorily with those predicted for 3-H^+^C_14_H_10_ (Figures 3e and S9f, in red), but align less well with other isomers of H^+^C_14_H_10_ (Figures 3 and S9). The two most intense features, observed at 1414.7/1410.6 and 1510.3 cm^–1^, correspond closely to scaled harmonic vibrational wavenumbers predicted at 1404 and 1514 cm^–1^ (both associated with C=C stretching modes). The reason for the splitting of 1414.7/1410.6 cm^–1^ is unknown, likely due to Fermi resonance with a combination or overtone band. Additionally, three medium-intense features were observed at 1628.7, 1587.0, and 1466.1 cm^–1^, showing close correlation to those predicted near 1638, 1579, and 1465 (all C=C stretching modes). For the characteristic CH_2_-stretching (ν_10_) mode, a band observed at 2840.9 cm^–1^, which decreased upon irradiation at 524 nm (Figure S6), aligns with the scaled harmonic vibrational wavenumber predicted at 2858 cm^–1^ (symmetric CH_2_-stretching mode). Furthermore, 15 weaker bands also match with theoretical predictions. The ν_23_ mode was predicted near 1320 cm^–1^ to be intense, whereas modes of ν_24_ and ν_25_ near 1296 and 1287 cm^–1^ were predicted to be weak. In contrast, observed features at 1307.5, 1293.4, 1290.2, and 1287.7 cm^–1^ have moderate intensities, indicating possible coupling among these modes. As summarized in Table 4, a total of 21 modes were identified for 3-H^+^C_14_H_10_. All modes predicted with IR intensity >20 km mol^–1^ except four were observed. A band predicted near 1362 cm^–1^ was interfered with by bands in groups X and Z and a band predicted near 857 cm^–1^ was interfered by the parent. Two bands predicted near 1238 and 639 cm^–1^ might be too weak to observe. Thus, the features in group Y are assigned to 3-H^+^C_14_H_10_. The mean absolute deviation between observed wavenumbers and scaled harmonic vibrational wavenumbers predicted for 3-H^+^C_14_H_10_ is calculated as 4.7 ± 4.8 cm^–1^. The largest deviation of 17.5 cm^–1^ is for ν_10_, which is typical for the CH_2_–stretching mode of protonated PAH. The anharmonic vibrational calculations yield a value of 2827 cm^–1^, suggesting that the true value might be smaller than the scaled harmonic vibrational wavenumbers.

**Table 4:** Comparison of Experimental Vibrational Wavenumbers and Relative IR Intensities of 3- and 4-H^+^C_14_H_10_ with Those Predicted with the B3YLP/6–311++G­(d,p) Method

3-H^+^C_14_H_10_ (group Y)						4-H^+^C_14_H_10_ (group Z)					
mode	sym.	calculation		*p*-H_2_		mode	sym.	calculation		*p*-H_2_	
*v*_10_	a′	2858.4^a^	(36)^b^	2840.9	(−)^c^	*v*_10_	a′	2878.8^a^	(13)^b^		
*v*_11_	a′	1637.9	(91)	1628.7	(31)^d^	*v*_11_	a′	1626.2	(114)	1624.3	(29)^d^
*v*_12_	a′	1606.8	(36)	1606.1	(12)	*v*_12_	a′	1613.7	(41)	1607.4	(7)
*v*_13_	a′	1579.0	(224)	1587.0	(35)	*v*_13_	a′	1588.3	(17)	1576.3?	(7)
*v*_14_	a′	1551.8	(49)	1549.7	(26)	*v*_14_	a′	1546.4	(32)	^e^	
*v*_16_	a′	1513.6	(230)	1510.3	(82)	*v*_15_	a′	1520.8	(81)	1520.3	(17)
*v*_17_	a′	1465.0	(87)	1466.1	(37)	*v*_16_	a′	1480.8	(228)	1478.2	(62)
*v*_18_	a′	1441.8	(14)	1441.9	(6)	*v*_17_	a′	1456.6	(58)	^e^	
*v*_20_	a′	1403.5	(161)	1414.7	(100)	*v*_18_	a′	1436.0	(55)	1435.1	(16)
				1410.6		*v*_19_	a′	1415.6	(23)	1412.4?	(17)
*v*_21_	a′	1362.4	(30)	^f^ *^,^* ^g^		*v*_20_	a′	1400.5	(106)	1403.8	(100)
*v*_22_	a′	1345.3	(96)	1343.2	(20)	*v*_21_	a′	1391.7	(225)	1401.1	(100)
*v*_23_	a′	1320.6	(158)	1307.5?	(10)	*v*_22_	a′	1357.2	(45)	1351.6?	(17)
*v*_24_	a′	1296.3	(4)	1293.4	(15)	*v*_23_	a′	1328.6	(53)	^h^	
				1290.2?	(23)	*v*_29_	a′	1179.1	(14)	1176.0?	(8)
*v*_25_	a′	1287.1	(9)	1287.7	(12)	*v*_55_	a″	875.4	(22)	871.9	(13)
*v*_26_	a′	1260.1	(41)	1258.5	(5)	*v*_56_	a″	810.3	(30)	809.2	(15)
*v*_27_	a′	1238.0	(22)	^i^		*v*_59_	a″	714.7	(48)	711.0	(24)
*v*_28_	a′	1221.8	(97)	1227.4	(28)						
*v*_30_	a′	1166.9	(42)	1162.3	(24)						
*v*_31_	a′	1158.2	(20)	1157.5	(14)						
*v*_36_	a′	914.4	(32)	923.4	(16)						
*v*_53_	a″	936.8	(22)	932.1	(14)						
*v*_54_	a″	880.7	(20)	880.0?	(7)						
*v*_55_	a″	857.1	(26)	^e^							
*v*_58_	a″	761.1	(48)	760.4	(10)						
*v*_60_	a″	639.1	(21)	^i^							

a Harmonic vibrational wavenumbers (cm^–1^) are scaled according to 0.9548x + 27.9 for wavenumbers >2000 cm^–1^ and 0.9804x + 2.33 for wavenumbers <2000 cm^–1^.

b Harmonic IR intensities (in km mol^–1^) are listed in parentheses. Only those with intensities >20 km mol^–1^ are listed.

c Interfered by groups X and W.

d Percentage integrated intensities of each isomer relative to the most intense band are listed in parentheses.

e Interfered by C_14_H_10_.

f Interfered by group X.

g Interfered by group Z.

h Interfered by group W.

i Too weak to be observed.

The observed wavenumbers and relative IR intensities of bands in group Z agree satisfactorily with those predicted for 4-H^+^C_14_H_10_ (Figures 3h and S10g, in blue), but do not match other isomers of H^+^C_14_H_10_ (Figures 3 and S10). The two most intense features, observed at 1403.8 and 1401.1 cm^–1^, closely correspond to the scaled harmonic vibrational wavenumbers predicted at 1401 and 1392 cm^–1^ (both associated with C=C stretching modes). Additionally, two medium-intensity bands, observed at 1624.3 and 1478.2 cm^–1^, align well with predictions near 1626 and 1481 cm^–1^ (both C=C stretching modes). Furthermore, 10 weaker features also match with theoretical predictions. A band observed at 1351.6 cm^–1^ is close to the band at 1351.1 cm^–1^ of group X, yet it can be distinguished upon irradiation at 463 nm, as the mixing ratio of group X decreased by only ∼ 4 ppb. As summarized in Table 4, a total of 14 bands were identified for 4-H^+^C_14_H_10_. All modes predicted with IR intensity >20 km mol^–1^ were observed, except for two bands that were predicted near 1546 and 1457 cm^–1^ but interfered by the parent, and one predicted near 1324 cm^–1^ but interfered by a feature in group W. Additionally, two bands at 1576.3 and 1176.0 cm^–1^ were observed despite their smaller predicted intensities of 17 and 14 km mol^–1^, respectively. Thus, the features in group Z are assigned to 4-H^+^C_14_H_10_. The mean absolute deviation between the observed and scaled harmonic vibrational wavenumbers for 4-H^+^C_14_H_10_ is 4.1 ± 3.3 cm^–1^. Because the CH_2_-stertching (ν_10_) mode near 2879 cm^–1^ was unidentified, the largest deviation of 12.0 cm^–1^ is for ν_13_, for which the observed band position at 1576.3 cm^–1^ was uncertain. Other bands deviate by <6.3 cm^–1^.

### 4.3 Absence of Features of 2-H^+^C_14_H_10_ and C_14_H_10_^+^

Computational predictions indicate that several features of 2-H^+^C_14_H_10_, located near 1628, 1619, 1600, 1478, 1468, 1370, 1332, and 1310 cm^–1^, exhibit IR intensities exceeding 90 km mol^–1^, with the most prominent feature appearing near 1600 cm^–1^ (IR intensity 225 km mol^–1^), as illustrated in Figures 3i and S3. However, upon closer examination, although some observed features are positioned near the predicted bands for 2-H^+^C_14_H_10_, their behaviors upon secondary irradiation are inconsistent and those features have been attributed to groups W, X, Y, and Z. Based on this analysis, we conclude that 2-H^+^C_14_H_10_ was produced in a negligible proportion. Isomer 2-H^+^C_14_H_10_ has the largest energy (∼8 kJ mol^–1^) among five low-lying isomers; energies of the other four isomers (9-, 1-, 3-, and 4-H^+^C_14_H_10_) are within 4 kJ mol^–1^.

After comparison with the gas-phase data^34^ and scaled harmonic vibrational wavenumbers predicted for C_14_H_10_^+^, we are confident that C_14_H_10_^+^ was not produced in significant amount in our experiments. In Figure S11, we also compare the predicted stick spectra of isomers of HC_14_H_10_ (monohydrogenated phenanthrene) with observed features of groups W–Z (assigned to H^+^C_14_H_10_). Features of H^+^C_14_H_10_, corresponding to downward bands in trace (c), are indicated with dashed lines with the same color-codes as those in other figures, while features of HC_14_H_10_, corresponding to upward bands in trace (c), are indicated with * marks. It is clear that predicted intense features of HC_14_H_10_ are in the region of 700–850 cm^–1^, inconsistent with observed features of groups W–Z. In these experiments, absorption bands of the hydrogenated species HC_14_H_10_ are relatively weak. We hence employed a different method to produce these hydrogenated species more efficiently to facilitate their spectral assignments, to appear in a forthcoming paper.

### 4.4 Formation Mechanism of H^+^C_14_H_10_ in solid *p*-H_2_

Electron bombardment of H_2_ ionizes the molecule (ionization potential 15.4 eV)^35^ to produce H_2_^+^; H_2_^+^ subsequently reacts with another H_2_, yielding H_3_^+^ and a hydrogen atom. Given that the proton affinity of C_14_H_10_ (∼829 kJ mol^–1^ at site 9) is significantly larger than that of H_2_ (422.3 kJ mol^–1^),^36^ H_3_^+^ readily transfers a proton to C_14_H_10_, forming H_2_ and H^+^C_14_H_10_ cations. These cations may be neutralized by electrons, resulting in the formation of HC_14_H_10_. Additionally, the reaction between H and C_14_H_10_ may also lead to the production of HC_14_H_10_. Thus, electron bombardment generates both H^+^C_14_H_10_ and HC_14_H_10_. After the electron-bombarded matrix was maintained in darkness, H^+^C_14_H_10_ might undergo reactions with trapped electrons to form HC_14_H_10_, while residual H atoms might react with C_14_H_10_ to yield HC_14_H_10_. Consequently, prolonged dark conditions led to an increase in the intensities of HC_14_H_10_ absorption bands, whereas those of H^+^C_14_H_10_ decrease, allowing distinct identification of features belonging to protonated and neutral species. This formation mechanism has been substantiated by previous electron bombardment experiments such as naphthalene,^15^ pyrene,^37^ coronene,^38^ ovalene,^39^ and corannulene.^23,40^ In this paper, we focus exclusively on the spectra of various isomers of H^+^C_14_H_10_, while the spectra of HC_14_H_10_ will be discussed separately in a forthcoming paper.

The proton transfer reactions, H_3_^+^ + *j*-C_14_H_10_ → *j*-H^+^C_14_H_10_ + H_2_, for the formation of 9-, 1-, 2-, 3-, and 4-H^+^C_14_H_10_ is exothermic, with respective energy releases of about 407, 404, 399, 403, and 403 kJ mol^–1^. The formation of all isomers of H^+^C_14_H_10_, except those protonated on the carbon of the fused ring (4a- and 8a-H^+^C_14_H_10_), should be feasible. However, our experimental observations indicate the presence of only four isomers, 9-, 1-, 3-, and 4-H^+^C_14_H_10_, while 2-H^+^C_14_H_10_ was absent. This discrepancy may arise from the fact that 2-H^+^C_14_H_10_ has an energy 8.4 kJ mol^–1^ higher than 9-H^+^C_14_H_10_, whereas the other observed isomers are within 4 kJ mol^–1^ of 9-H^+^C_14_H_10_. Although the estimated nixing ratios indicate that 1-H^+^C_14_H_10_ has the smallest mixing ratio, given the inherent large uncertainties in predicted IR intensities and integrated absorbance of observed bands, which directly impact the estimation of mixing ratios for each species, we conclude that the mixing ratios of all four observed isomers of H^+^C_14_H_10_ are comparable (Table 2).

### 4.5 Photolytic Behavior of Observed Protonated Phenanthrene

Irradiation in the 619–463 nm range provides energies of 193–258 kJ mol^–1^, insufficient to deprotonate H^+^C_14_H_10_, which requires ∼ 407 kJ mol^–1^. However, this energy is significantly greater than that needed for proton-transfer to adjacent carbon atoms, which occurs at less than 98 kJ mol^–1^ (Table 1 and Figure S2). Even though we could not calculate the proton-transfer pathways upon electronic excitation, the observed correlation between the decrease in the mixing ratio of one isomer and the increase in the mixing ratio of another isomer (Table 2) strongly suggests that proton transfer to the neighboring carbon site takes place upon irradiation within the 619–463 nm range.

The vertical excitation spectra of 9-, 1-, 2-, 3-, and 4-H^+^C_14_H_10_, predicted in the range 400–700 nm using the TD-B3LYP/6–311++G­(d,p) method, are illustrated in Figure S12b. Corresponding wavelengths and oscillator strengths are detailed in Table S6. These calculated UV spectra provide insights into the behavior of each species during secondary irradiation. The experimental electronic absorption spectra of various isomers of protonated phenanthrenes in solid Ne reported by Garkusha et al.^41^ is depicted in Figure S12a for comparison. The transition origins appear to be the most intense bands of each progression. In general, experimental results are in agreement with calculated vertical transitions listed in Table S6 (with deviations within 14 nm, except for the absorption to the first excited state of 2-H^+^C_14_H_10_, which deviates by ∼ 28 nm). However, the experimental progression starting at 596.6 nm might have to be assigned to 4-H^+^C_14_H_10_ instead of 9-H^+^C_14_H_10_, according to quantum-chemical calculations and our experimental observations.

At 619 nm, only 4-H^+^C_14_H_10_ (group Z) was predicted to exhibit significant absorption, explaining that 4-H^+^C_14_H_10_ is the only species that decreased in mixing ratio. This decline is accompanied by a corresponding increase in the mixing ratio of 3-H^+^C_14_H_10_ (group Y), suggesting that irradiation at this wavelength induce proton transfer from position 4 to position 3. Upon irradiation at 544 nm, the mixing ratio of 4-H^+^C_14_H_10_ (group Z) continued to decrease, along with 1-H^+^C_14_H_10_ (group X), which has a predicted absorption maximum near 523 nm. The mixing ratio of 3-H^+^C_14_H_10_ (group Y) continued to increase. Although the predicted vertical absorption of 4-H^+^C_14_H_10_ is near 618 and 421 nm (Table S6), the experimental progression for the system originated near 596.6 nm (originally assigned to 9-H^+^C_14_H_10_ but should be 4-H^+^C_14_H_10_) extends beyond 544 nm, suggesting that 4-H^+^C_14_H_10_ might be excited at 544 nm, explaining the decrease of features in group Z.

When irradiated at 524 nm, both 3-H^+^C_14_H_10_ (group Y) and 1-H^+^C_14_H_10_ (group X) showed a reduction in mixing ratio, whereas 4-H^+^C_14_H_10_ (group Z) increased. 1-H^+^C_14_H_10_ was predicted to absorb near 523 nm. The predicted vertical excitation of 3-H^+^C_14_H_10_ is near 462 nm; however, this band may have been overestimated in energy or extend to 524 nm. At 463 nm, 1-H^+^C_14_H_10_ (group X) continued to decrease, while the trends of 3-H^+^C_14_H_10_ (group Y) and 4-H^+^C_14_H_10_ (group Z) reversed compared to their behavior at 524 nm. The decrease in 4-H^+^C_14_H_10_ might be attributed to an intense band predicted near 421 nm for H^+^C_14_H_10_. Bands peaking near 471 and 530 nm were predicted for 9-H^+^C_14_H_10_ (group W), but its mixing ratio remained largely unchanged upon irradiation at wavelengths 619–524 nm, exhibiting only a ∼ 10% decrease at 463 nm. This behavior suggests that, at position 9, proton transfer to positions 1–4 is more challenging, whereas proton transfer to position 10 results in the same isomer.

### 4.6 Implications to UIR Identification

Figure 4 compares the experimental stick IR spectra of 9-, 1-, 3-, and 4-H^+^C_14_H_10_ with the unidentified infrared (UIR) bands observed in the Orion Bar PDR.^1^ The UIR bands are considered to be UV-induced IR emission spectra. Considering vibrational anharmonicity, red shifts of these emission features from IR absorption bands observed at ambient temperature in laboratories are expected.^42^ To simulate the spectra, the observed stick spectra for each species were convoluted using a Lorentzian function (full-width at half-maximum = 20 cm^–1^). Additionally, to account for deviations caused by unobserved features due to spectral interferences, convoluted spectra based on scaled harmonic vibrational wavenumbers and harmonic IR intensities are presented in corresponding light-colored plots. Overall, the predicted spectra show satisfactory agreement with the experimental observations.

**Figure 4:**
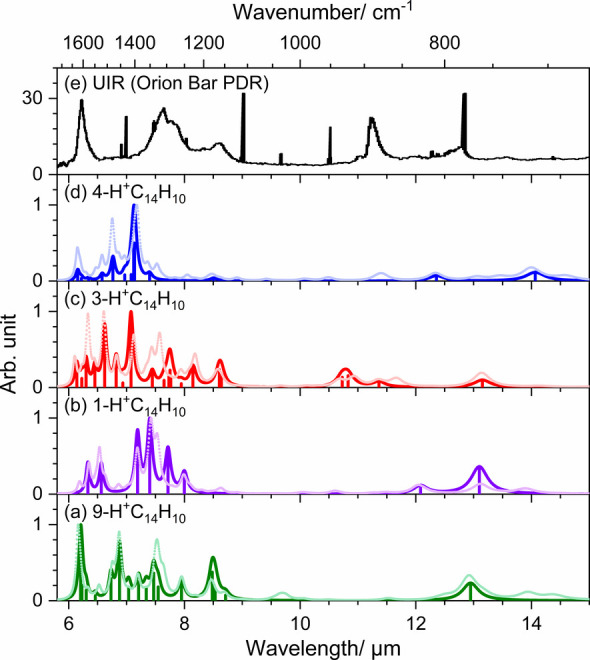
Comparison of UIR emission bands with IR spectra of H^+^C_14_H_10_. (a)–(d) IR spectra of 9-, 1-, 3-, and 4-H^+^C_14_H_10_. The observed sticks are convoluted with a Lorentzian function (FWMH = 20 cm^–1^) to produce spectra with the same color. For each species, the spectra with a light color is the scaled harmonic vibrational spectrum convoluted with a Lorentzian function (FWMH = 20 cm^–1^). (e) UIR emission spectrum from Orion Bar PDR. Reproduced from Peeters et al.^3^ Copyright 2021 American Chemical Society.

The characteristic spectral features of these monoprotonated C_14_H_10_ species include intense absorption bands in region 6–8 μm (primarily due to C=C stretching and in-plane CH-bending modes) and some weaker bands near 13 μm (out-of-plane CH-bending mode). However, these bands do not match the UIR bands, suggesting that protonated phenanthrene is unlikely to be a significant carrier of the UIR bands. This conclusion is plausible, as H^+^C_14_H_10_ is too small to withstand the intense UV radiation in interstellar environments. Nevertheless, our findings reinforce previous studies on protonated naphthalene,^15^ pyrene,^37^ coronene,^38^ and ovalene,^39^ indicating that protonation of PAH enhances spectral features in the 6–8 μm region.

## 5 Conclusions

Electron bombardment during the deposition of a mixture of C_14_H_10_ and *p*-H_2_ onto a cold substrate led to the formation of four isomers of protonated phenanthrene, 9-, 1-, 3-, and 4-H^+^C_14_H_10_, among other hydrogenated species. The IR features of these isomers diminished after prolonged storage of the matrix in darkness due to neutralization with trapped electrons. Based on their behaviors upon irradiation at 619, 544, 524, and 463 nm, these features were classified into four distinct groups. Assignments of these features to 9-, 1-, 3-, and 4-H^+^C_14_H_10_ were confirmed through comparison with scaled harmonic vibrational wavenumbers and harmonic IR intensities predicted using the B3LYP/6–311++G­(d,p) method. The IR spectra of these four monoprotonated phenanthrene isomers are previously unreported. They might provide important information for probing these protonated species that might play a key role in the bottom-up mechanism of the formation of PAH. Furthermore, the photolytic behavior of each isomer at various wavelengths has been rationalized based on electronic absorption predicted by quantum-chemical computations and proton transfer to a neighboring site. The IR spectra of 9-, 1-, 3-, and 4-H^+^C_14_H_10_ suggest that these species are unlikely to serve as major carriers of the UIR bands.

## Supplementary Material



## Notes

Scaled harmonic vibrational wavenumbers and IR intensities of 9-, 1-, 2-, 3-, 4-, 4a-, and 8a-H^+^C_14_H_10_; comparison of observed vibrational wavenumbers of C_14_H_10_ in solid *p*-H_2_ and Ar with computed values; estimated mixing ratio of C_14_H_10_ and 9-, 1-, 3-, and 4-H^+^C_14_H_10_; predicted vertical excitation wavelengths and oscillator strengths of electronic excitation of C_14_H_10_ and 9-, 1-, 2-, 3-, 4-, 4a-, and 8a-H^+^C_14_H_10_ (6 tables); geometries and relative energies of seven possible isomers of H^+^C_14_H_10_; potential energy scheme for proton transfer of various isomers of H^+^C_14_H_10_; computed stick spectra of C_14_H_10_ and isomers of H^+^C_14_H_10_; comparison of the IR spectra of C_14_H_10_ in solid *p*-H_2_ and Ar with the computed stick spectrum of C_14_H_10_; infrared spectra in the range 975–1640 cm^–1^ of an electron-bombarded C_14_H_10_/*p*-H_2_ matrix after each experimental step; comparison of observed IR spectra in region 2650–3150 cm^–1^ with stick spectra of seven isomers of H^+^C_14_H_10_; comparison of bands in groups W, X, Y, and Z in region 575–1640 cm^–1^ with stick spectra of seven isomers of H^+^C_14_H_10_; calculated vertical electronic excitation spectra of 9-, 1-, 2-, 3-, and 4-H^+^C_14_H_10_ (11 figures) (PDF)
