# Supplementary material for: Infrared Study of Electron-bombarded Phenanthrene (C14H10)/Para-H2 Matrices: Isomers of Protonated Phenanthrene (1‑, 3‑, 4‑, and 9‑H+C14H10)
Source: J Phys Chem A. 2025 Sep 29;129(40):9260–70. doi: 10.1021/acs.jpca.5c04756 (PMC12516719; doi:10.1021/acs.jpca.5c04756)
Supplement: Supplementary file 1 [file jp5c04756_si_001.pdf]

Supporting Information

Infrared Study of Electron-bombarded Phenanthrene ($C_{14}H_{10}$)/*Para*-H_2 Matrices: Isomers of Protonated Phenanthrene (1-, 3-, 4-, and 9-$H^+C_{14}H_{10}$)

Jun-Ying Feng[†] and Yuan-Pern Lee[†,‡*]

[†]*Department of Applied Chemistry and Institute of Molecular Science, National Yang Ming Chiao Tung University, Hsinchu 300093, Taiwan*

[‡]*Center for Emergent Functional Matter Science, National Yang Ming Chiao Tung University, Hsinchu 300093, Taiwan*

**E-mail: yplee@nycu.edu.tw* (Y.-P. Lee)

Table of Contents

Table S1. Scaled harmonic and anharmonic vibrational wavenumbers and IR intensities of 9-, 1-, and 2-$H^+C_{14}H_{10}$ predicted with the B3LYP/6-311++G(d,p) method S1

Table S2. Scaled harmonic and anharmonic vibrational wavenumbers and IR intensities of 3- and 4-$H^+C_{14}H_{10}$ predicted with the B3LYP/6-311++G(d,p) method S5

Table S3. Scaled harmonic vibrational wavenumbers and IR intensities of 4a- and 8a-$H^+C_{14}H_{10}$ predicted with the B3LYP/6-311++G(d,p) method S8

Table S4. Comparison of observed vibrational wavenumbers of $C_{14}H_{10}$ in solid *p*-H_2 and Ar with predicted scaled harmonic vibrational wavenumbers and IR intensities of $C_{14}H_{10}$ with various methods S10

Table S5. Summary of estimated mixing ratios of each species in the electron-bombarded $C_{14}H_{10}$/*p*-H_2 matrix experiment S13

Table S6. Vertical excitation wavelengths and oscillator strengths of electronic excitation of $C_{14}H_{10}$ and 9-, 1-, 2-, 3-, 4-, 4a-, and 8a-$H^+C_{14}H_{10}$ predicted with the TD-B3LYP/6-311++G(d,p) method S14

Figure S1. Geometries and relative energies of seven possible isomers of $H^+C_{14}H_{10}$ S16

Figure S2. Potential energy scheme for the proton transfer of various isomers of $H^+C_{14}H_{10}$ S17

Figure S3. Computed stick spectra of $C_{14}H_{10}$ and isomers of $H^+C_{14}H_{10}$ S18

Figure S4. Comparison of the IR absorption spectra of $C_{14}H_{10}$ isolated in solid *p*-H_2 and Ar with the computed stick spectrum of $C_{14}H_{10}$ S19

Figure S5. Infrared spectra (in region 975–1640 cm^{-1}) of an electron-bombarded $C_{14}H_{10}$/*p*-H_2 matrix after each experimental step S20

Figure S6. Comparison of observed IR spectra in region 2650–3150 cm^{-1} with stick spectra of seven isomers of $H^+C_{14}H_{10}$ S22

Figure S7. Comparison of lines in group W in region 575−1640 cm^{-1} with stick spectra of seven isomers of $H^+C_{14}H_{10}$ S23

Figure S8. Comparison of lines in group X in region 575−1640 cm^{-1} with stick spectra of seven isomers of $H^+C_{14}H_{10}$ S25

Figure S9. Comparison of lines in group Y in region 575−1640 cm^{-1} with stick spectra of seven isomers of $H^+C_{14}H_{10}$ S27

Figure S10. Comparison of lines in group Z in region 575−1640 cm^{-1} with stick spectra of seven isomers of $H^+C_{14}H_{10}$ S29

Figure S11. Comparison of lines of $H^+C_{14}H_{10}$ and $HC_{14}H_{10}$ in region 575−1640 cm^{-1} with stick spectra of isomers of $HC_{14}H_{10}$ S31

Figure S12. Experimental UV spectra and calculated vertical electronic excitation spectra of 9-, 1-, 2-, 3-, and 4-$H^+C_{14}H_{10}$ S33

Reference. S34

Table S1. Scaled Harmonic and Anharmonic Vibrational Wavenumbers and IR Intensities of 9-, 1-, and 2-$H^+C_{14}H_{10}$ Predicted with the B3LYP/6-311++G(d,p) Method

mode	sym.	9-$H^+C_{14}H_{10}$				1-$H^+C_{14}H_{10}$				2-$H^+C_{14}H_{10}$			
		calculation[a]				calculation[a]				calculation[a]			
		harmonic		anharmonic		harmonic		anharmonic		harmonic		anharmonic	
ν_1	a′	3110.8	(0.7)	3102.1	(0.7)	3092.6	(0.5)	3084.8	(0.1)	3112.3	(0.6)	3109.2	(1.4)
ν_2	a′	3094.9	(0.7)	3117.7	(1.2)	3089.1	(0.6)	3034.3	(1.3)	3095.9	(0.5)	3107.2	(1.5)
ν_3	a′	3092.3	(0.1)	3106.2	(0.3)	3085.1	(1.8)	3069.1	(1.1)	3084.9	(0.6)	3087.1	(2.0)
ν_4	a′	3084.5	(0.7)	3079.6	(1.4)	3077.1	(1.0)	3108.7	(1.8)	3081.5	(0.6)	3064.3	(3.0)
ν_5	a′	3074.0	(0.4)	3068.0	(1.1)	3072.6	(0.4)	3056.3	(0.9)	3076.9	(0.3)	3067.8	(0.2)
ν_6	a′	3072.0	(0.0)	3059.2	(1.8)	3070.6	(0.5)	3088.6	(0.5)	3073.9	(0.9)	3055.9	(0.2)
ν_7	a′	3068.1	(0.2)	3041.2	(1.3)	3068.6	(2.2)	3041.4	(5.2)	3068.1	(0.3)	3047.5	(0.5)
ν_8	a′	3057.8	(1.4)	3019.8	(1.6)	3065.7	(0.6)	3044.4	(0.8)	3066.2	(0.2)	3081.3	(0.8)
ν_9	a′	3052.9	(0.4)	3031.4	(0.4)	3059.0	(0.1)	3024.2	(0.9)	3058.1	(0.3)	3035.0	(0.5)
ν_{10}	a′	2864.3	(30.7)	2827.8	(28.4)	2873.0	(30.7)	2841.9	(26.3)	2859.4	(49.1)	2830.7	(18.6)
ν_{11}	a′	1623.1	(221.8)	1612.9	(50.7)	1616.4	(49.5)	1610.2	(12.8)	1627.9	(105.8)	1619.6	(47.8)
ν_{12}	a′	1611.8	(54.7)	1603.8	(12.6)	1610.3	(1.0)	1603.7	(11.3)	1618.7	(97.3)	1610.2	(46.7)
ν_{13}	a′	1579.0	(24.8)	1572.3	(13.1)	1575.0	(135.4)	1577.2	(70.1)	1600.1	(225.2)	1594.5	(51.0)
ν_{14}	a′	1551.6	(5.1)	1543.5	(2.7)	1546.7	(28.8)	1536.6	(0.1)	1551.4	(22.9)	1544.6	(20.0)
ν_{15}	a′	1532.5	(39.2)	1524.0	(18.5)	1530.8	(201.4)	1525.3	(85.1)	1533.0	(10.2)	1525.7	(4.1)
ν_{16}	a′	1481.1	(91.4)	1480.7	(49.8)	1507.9	(51.9)	1498.2	(50.9)	1478.1	(107.8)	1474.1	(26.6)
ν_{17}	a′	1465.2	(23.8)	1460.0	(8.0)	1457.5	(31.2)	1452.9	(9.9)	1467.8	(108.2)	1464.1	(43.8)
ν_{18}	a′	1454.6	(196.5)	1450.7	(98.3)	1441.3	(1.2)	1440.9	(2.9)	1440.9	(10.2)	1439.1	(7.7)
ν_{19}	a′	1420.2	(15.8)	1409.8	(5.6)	1424.5	(14.8)	1418.0	(8.6)	1420.8	(3.9)	1417.2	(0.5)
ν_{20}	a′	1389.8	(72.1)	1380.3	(37.3)	1391.3	(200.4)	1394.1	(4.0)	1406.8	(42.0)	1400.4	(19.5)
ν_{21}	a′	1363.8	(12.8)	1353.1	(7.7)	1360.2	(6.7)	1351.3	(4.3)	1370.6	(113.6)	1366.7	(76.8)

ν_{22}	a′	1333.5	(89.0)	1324.8	(34.4)	1347.4	(309.1)	1348.1	(241.2)	1356.0	(14.7)	1339.8	(2.8)
ν_{23}	a′	1326.5	(112.1)	1299.1	(45.6)	1327.3	(221.5)	1307.2	(86.4)	1331.5	(90.8)	1329.9	(37.4)
ν_{24}	a′	1308.9	(71.6)	1309.3	(72.9)	1303.5	(6.3)	1300.3	(5.5)	1309.6	(127.2)	1289.4	(123.4)
ν_{25}	a′	1284.4	(2.0)	1285.7	(1.3)	1283.8	(11.2)	1284.7	(8.8)	1283.2	(11.4)	1285.9	(7.3)
ν_{26}	a′	1257.5	(69.8)	1256.7	(50.0)	1251.7	(80.6)	1252.5	(53.4)	1258.3	(26.4)	1258.6	(18.7)
ν_{27}	a′	1220.4	(5.7)	1219.6	(2.7)	1234.0	(20.6)	1232.7	(13.5)	1221.3	(20.7)	1220.3	(11.7)
ν_{28}	a′	1188.0	(0.5)	1195.0	(0.3)	1204.2	(9.2)	1203.7	(4.0)	1207.5	(1.3)	1208.9	(2.3)
ν_{29}	a′	1182.2	(45.3)	1186.2	(4.1)	1181.0	(4.3)	1189.0	(3.6)	1180.5	(12.9)	1188.3	(13.5)
ν_{30}	a′	1177.8	(20.8)	1180.1	(102.0)	1170.3	(1.1)	1175.6	(1.3)	1167.9	(23.1)	1175.6	(9.2)
ν_{31}	a′	1150.0	(19.8)	1153.7	(24.5)	1158.8	(24.1)	1164.9	(21.3)	1159.4	(6.6)	1164.0	(4.6)
ν_{32}	a′	1107.4	(1.1)	1108.7	(0.2)	1121.8	(4.4)	1123.8	(1.0)	1111.1	(11.2)	1115.6	(4.4)
ν_{33}	a′	1048.6	(3.9)	1052.4	(4.3)	1064.1	(1.9)	1067.2	(0.9)	1042.6	(5.7)	1044.9	(3.9)
ν_{34}	a′	1036.2	(14.1)	1037.8	(13.5)	1041.8	(1.6)	1044.6	(1.6)	1027.1	(14.9)	1028.4	(18.0)
ν_{35}	a′	1026.5	(14.2)	1028.0	(10.7)	995.8	(5.9)	999.0	(3.8)	990.0	(8.0)	994.3	(6.8)
ν_{36}	a′	994.6	(4.1)	1001.3	(3.8)	942.9	(15.1)	948.0	(10.2)	923.3	(27.9)	925.6	(24.7)
ν_{37}	a′	852.3	(0.3)	861.8	(0.4)	868.5	(2.2)	874.1	(1.8)	853.5	(2.0)	857.3	(2.2)
ν_{38}	a′	807.4	(2.6)	813.3	(1.0)	823.0	(4.6)	826.8	(4.7)	814.0	(7.3)	815.4	(7.7)
ν_{39}	a′	696.2	(11.7)	699.8	(10.5)	707.2	(0.1)	711.7	(0.0)	701.9	(1.2)	705.5	(0.7)
ν_{40}	a′	690.8	(4.3)	698.1	(2.2)	687.0	(1.7)	690.8	(1.6)	691.1	(8.2)	694.8	(7.2)
ν_{41}	a′	613.8	(2.7)	618.2	(2.5)	603.9	(0.7)	606.5	(0.7)	601.4	(8.4)	605.5	(6.0)
ν_{42}	a′	538.8	(0.2)	545.4	(0.2)	534.3	(17.3)	537.0	(15.1)	537.3	(2.8)	540.4	(2.7)
ν_{43}	a′	490.1	(2.4)	494.8	(2.4)	488.7	(1.9)	491.3	(1.7)	488.0	(0.6)	491.2	(0.5)
ν_{44}	a′	431.7	(0.8)	433.4	(0.8)	436.5	(3.8)	437.7	(2.5)	435.7	(0.6)	436.7	(0.6)
ν_{45}	a′	395.4	(6.6)	394.9	(3.2)	395.9	(3.8)	397.1	(3.7)	404.2	(4.2)	406.8	(3.1)
ν_{46}	a′	244.5	(1.9)	239.5	(1.1)	238.0	(1.5)	239.1	(1.2)	248.2	(1.2)	245.6	(1.2)
ν_{47}	a″	2868.3	(5.2)	2814.6	(5.3)	2880.0	(5.3)	2832.6	(3.9)	2858.9	(7.1)	2810.3	(6.3)
ν_{48}	a″	1160.5	(1.2)	1145.2	(1.0)	1163.7	(1.1)	1153.0	(2.1)	1144.4	(0.0)	1129.4	(0.0)

ν_{49}	a″	1015.8	(0.5)	1019.3	(0.7)	1008.4	(0.5)	980.5	(0.0)	1009.8	(0.5)	1009.0	(0.3)
ν_{50}	a″	1005.9	(0.3)	1002.5	(0.0)	1004.5	(0.4)	1012.7	(0.0)	998.6	(1.0)	1011.7	(0.2)
ν_{51}	a″	989.4	(0.6)	994.0	(1.5)	995.9	(0.8)	1017.6	(3.2)	984.9	(0.8)	995.5	(0.0)
ν_{52}	a″	971.1	(0.0)	972.3	(0.1)	984.2	(2.1)	1124.1	(13.8)	972.7	(1.9)	975.3	(1.9)
ν_{53}	a″	951.9	(2.1)	957.6	(0.8)	968.2	(0.2)	963.5	(3.6)	933.2	(20.2)	937.0	(22.9)
ν_{54}	a″	878.0	(0.0)	879.2	(1.5)	889.1	(3.8)	789.2	(72.9)	878.4	(10.1)	871.6	(3.8)
ν_{55}	a″	868.2	(6.5)	897.0	(2.3)	871.8	(4.0)	930.6	(75.1)	816.0	(13.9)	810.2	(11.6)
ν_{56}	a″	799.2	(9.3)	782.6	(47.4)	830.3	(39.9)	822.9	(51.7)	794.0	(16.4)	989.9	(0.4)
ν_{57}	a″	774.1	(76.2)	762.1	(2.6)	762.3	(46.7)	802.4	(37.6)	791.3	(10.4)	787.0	(15.2)
ν_{58}	a″	759.7	(14.6)	825.0	(5.3)	742.3	(2.8)	897.1	(15.8)	752.2	(53.2)	751.7	(50.3)
ν_{59}	a″	717.6	(23.2)	721.5	(53.4)	719.8	(24.6)	425.6	(16.3)	728.0	(9.6)	731.4	(12.6)
ν_{60}	a″	670.4	(1.5)	667.7	(0.0)	628.5	(8.8)	595.2	(79.8)	635.2	(4.1)	633.8	(4.5)
ν_{61}	a″	519.7	(3.2)	513.9	(2.7)	500.2	(4.1)	455.6	(17.1)	536.5	(0.2)	539.0	(0.2)
ν_{62}	a″	466.8	(1.0)	454.9	(1.4)	490.3	(1.4)	567.6	(24.2)	485.4	(1.6)	475.6	(1.6)
ν_{63}	a″	431.0	(0.0)	427.2	(0.0)	430.2	(2.7)	290.5	(22.3)	419.3	(12.5)	412.9	(13.6)
ν_{64}	a″	415.3	(7.8)	408.2	(8.2)	386.2	(0.2)	384.5	(11.1)	368.1	(1.3)	361.8	(1.0)
ν_{65}	a″	266.4	(1.1)	270.0	(0.0)	281.9	(10.6)	328.7	(81.1)	297.6	(2.4)	300.0	(1.8)
ν_{66}	a″	219.8	(3.3)	217.0	(4.0)	214.6	(4.1)	181.7	(10.0)	220.4	(4.3)	217.6	(4.9)
ν_{67}	a″	176.5	(8.7)	175.0	(9.2)	159.5	(0.5)	283.6	(0.0)	188.7	(0.2)	187.2	(0.2)
ν_{68}	a″	90.7	(0.2)	87.6	(0.2)	100.4	(2.0)	102.7	(1.4)	91.1	(0.8)	86.6	(1.3)
ν_{69}	a″	69.1	(0.4)	65.5	(0.2)	68.5	(0.3)	40.9	(4.3)	79.9	(2.9)	80.3	(2.4)
			overtone/combination mode[b]				overtone/combination mode[b]				overtone/combination mode[b]		
			$2\nu_{38}$	1626.6	(16.9)		$\nu_{39} + \nu_{38}$	1537.8	(15.8)		$\nu_{22} + \nu_{14}$	2885.3	(17.3)
			$\nu_{44} + \nu_{29}$	1619.4	(62.9)		$\nu_{40} + \nu_{37}$	1566.6	(16.6)		$\nu_{41} + \nu_{34}$	1632.9	(27.8)
			$\nu_{44} + \nu_{33}$	1485.4	(20.6)		$\nu_{43} + \nu_{37}$	1366.7	(64.8)		$\nu_{43} + \nu_{32}$	1606.1	(41.5)
			$\nu_{46} + \nu_{20}$	1625.2	(32.1)		$\nu_{44} + \nu_{29}$	1625.6	(30.5)		$\nu_{43} + \nu_{35}$	1484.8	(24.0)
			$\nu_{58} + \nu_{55}$	1659.6	(15.4)		$\nu_{45} + \nu_{35}$	1395.9	(60.4)		$\nu_{44} + \nu_{30}$	1609.7	(95.0)

$\nu_{60} + \nu_{53}$	1627.6	(35.7)	$\nu_{46} + \nu_{22}$	1588.0	(24.0)	$\nu_{44} + \nu_{33}$	1481.1	(42.3)
$\nu_{61} + \nu_{56}$	1318.8	(32.7)	$\nu_{46} + \nu_{31}$	1404.5	(32.7)	$\nu_{46} + \nu_{21}$	1607.0	(32.0)
$\nu_{62} + \nu_{48}$	1600.8	(76.5)	$\nu_{58} + \nu_{56}$	1575.1	(17.9)	$\nu_{46} + \nu_{22}$	1588.0	(20.0)
$\nu_{62} + \nu_{50}$	1456.0	(36.2)	$\nu_{59} + \nu_{56}$	1521.1	(19.7)	$\nu_{46} + \nu_{27}$	1465.4	(35.2)
$\nu_{62} + \nu_{51}$	1447.9	(46.8)	$\nu_{60} + \nu_{57}$	1309.5	(22.6)	$\nu_{59} + \nu_{54}$	1654.5	(31.0)
$\nu_{62} + \nu_{54}$	1338.5	(31.7)	$\nu_{61} + \nu_{54}$	1338.3	(38.4)	$\nu_{62} + \nu_{50}$	1485.9	(22.6)
$\nu_{64} + \nu_{57}$	1186.9	(23.6)	$\nu_{62} + \nu_{56}$	1328.4	(23.5)	$\nu_{65} + \nu_{48}$	1427.8	(15.4)
			$\nu_{64} + \nu_{49}$	1380.2	(101.5)			
			$\nu_{66} + \nu_{48}$	1379.3	(23.1)			
			$\nu_{67} + \nu_{48}$	1380.8	(57.6)			

[a]Harmonic vibrational wavenumbers are scaled according to $0.9548x + 27.9$ for wavenumbers > 2000 cm^{-1} and $0.9804x + 2.33$ for wavenumbers < 2000 cm^{-1}. Harmonic IR intensities (in km mol^{-1}) are listed in parentheses. [b]Overtone and combination bands with the intensity > 15 km mol^{-1} are listed.

Table S2. Scaled Harmonic and Anharmonic Vibrational Wavenumbers and IR Intensities of 3- and 4-$H^+C_{14}H_{10}$ Predicted with the B3LYP/6-311++G(d,p) Method

mode	sym.	3-$H^+C_{14}H_{10}$				4-$H^+C_{14}H_{10}$			
		calculation[a]				calculation[a]			
		harmonic		anharmonic		harmonic		anharmonic	
ν_1	a′	3090.5	(0.8)	3047.9	(0.5)	3090.3	(0.6)	3087.7	(1.2)
ν_2	a′	3085.5	(1.3)	3083.9	(1.8)	3089.3	(1.0)	3101.2	(1.3)
ν_3	a′	3082.1	(0.2)	3073.3	(0.7)	3081.4	(1.9)	3081.6	(3.7)
ν_4	a′	3079.5	(0.1)	3066.2	(0.2)	3080.5	(0.4)	3093.2	(1.3)
ν_5	a′	3076.0	(0.8)	3097.9	(2.2)	3073.0	(0.0)	3066.7	(1.2)
ν_6	a′	3070.0	(0.0)	3086.2	(4.1)	3068.7	(1.0)	3061.9	(0.6)
ν_7	a′	3066.5	(1.4)	3017.6	(0.6)	3067.6	(0.2)	3044.7	(1.3)
ν_8	a′	3066.3	(0.2)	3038.0	(1.3)	3065.7	(0.4)	3054.1	(2.2)
ν_9	a′	3059.6	(0.2)	3042.8	(0.2)	3055.5	(0.9)	3032.7	(0.9)
ν_{10}	a′	2858.4	(35.5)	2827.2	(22.7)	2878.8	(12.8)	2847.9	(6.8)
ν_{11}	a′	1637.9	(91.4)	1626.6	(0.0)	1626.2	(113.9)	1618.3	(42.0)
ν_{12}	a′	1606.8	(36.0)	1599.9	(6.8)	1613.7	(40.9)	1605.7	(14.6)
ν_{13}	a′	1579.0	(224.0)	1574.9	(45.5)	1588.3	(16.7)	1577.6	(1.0)
ν_{14}	a′	1551.8	(49.3)	1539.2	(32.5)	1546.4	(31.9)	1541.6	(10.4)
ν_{15}	a′	1524.1	(12.2)	1516.6	(10.9)	1520.8	(80.9)	1513.5	(24.6)
ν_{16}	a′	1513.6	(229.8)	1504.2	(6.0)	1480.8	(227.9)	1472.5	(39.8)
ν_{17}	a′	1465.0	(87.2)	1462.5	(28.4)	1456.6	(58.3)	1450.8	(41.5)
ν_{18}	a′	1441.8	(14.3)	1441.6	(5.6)	1436.0	(54.7)	1433.9	(9.6)
ν_{19}	a′	1428.5	(0.7)	1427.7	(3.2)	1415.6	(23.3)	1409.9	(8.0)
ν_{20}	a′	1403.5	(160.7)	1400.5	(178.0)	1400.5	(105.9)	1394.6	(88.8)
ν_{21}	a′	1362.4	(30.4)	1348.8	(32.1)	1391.7	(225.0)	1386.7	(63.6)
ν_{22}	a′	1345.3	(95.9)	1332.0	(0.9)	1357.2	(44.5)	1348.2	(24.5)
ν_{23}	a′	1320.6	(157.7)	1307.3	(41.6)	1328.6	(52.5)	1307.8	(3.5)
ν_{24}	a′	1296.3	(3.6)	1292.5	(56.5)	1311.7	(6.4)	1304.2	(1.5)
ν_{25}	a′	1287.1	(8.8)	1283.3	(1.6)	1275.6	(5.9)	1277.0	(4.9)
ν_{26}	a′	1260.1	(41.3)	1261.8	(21.6)	1242.3	(19.5)	1242.7	(9.8)
ν_{27}	a′	1238.0	(22.2)	1237.2	(13.0)	1221.0	(4.7)	1220.0	(2.4)
ν_{28}	a′	1221.8	(97.1)	1226.9	(33.6)	1187.1	(9.8)	1188.5	(8.8)
ν_{29}	a′	1179.3	(5.4)	1187.1	(6.5)	1179.1	(13.9)	1186.9	(14.0)
ν_{30}	a′	1166.9	(41.5)	1170.8	(31.0)	1175.1	(5.1)	1179.9	(10.4)
ν_{31}	a′	1158.2	(19.5)	1164.7	(8.6)	1160.7	(7.6)	1165.7	(5.2)
ν_{32}	a′	1115.3	(2.1)	1119.0	(1.5)	1122.3	(11)	1128.4	(5.3)
ν_{33}	a′	1046.1	(0.2)	1051.4	(0.0)	1061.8	(6.9)	1066.0	(5.0)
ν_{34}	a′	1034.6	(4.3)	1036.4	(4.1)	1033.7	(1.4)	1036.0	(0.9)
ν_{35}	a′	989.3	(3.9)	991.7	(3.4)	993.4	(5.4)	1001.1	(3.7)

ν_{36}	a′	914.4	(31.7)	922.0	(9.5)	953.3	(13.1)	958.1	(8.6)
ν_{37}	a′	862.2	(1.1)	865.3	(1.2)	845.8	(0.1)	850.5	(0.3)
ν_{38}	a′	816.7	(1.5)	821.4	(1.7)	823.2	(0.8)	825.0	(1.1)
ν_{39}	a′	707.6	(3.5)	711.6	(3.0)	707.5	(3.2)	710.7	(1.5)
ν_{40}	a′	696.2	(0.4)	699.0	(0.2)	685.5	(17.2)	688.2	(13.2)
ν_{41}	a′	603.1	(4.2)	607.3	(3.9)	599.1	(0.7)	600.2	(0.5)
ν_{42}	a′	539.8	(5.7)	541.5	(5.2)	534.2	(5.5)	537.3	(5.1)
ν_{43}	a′	492.9	(6.9)	496.8	(6.1)	489.4	(1.6)	494.0	(1.3)
ν_{44}	a′	434.9	(0.9)	435.8	(0.9)	436.4	(0.1)	437.8	(0.2)
ν_{45}	a′	398.1	(2.8)	399.2	(2.8)	402.9	(10.9)	404.3	(10.5)
ν_{46}	a′	239.2	(0.4)	236.9	(0.4)	231.9	(1.1)	232.1	(1.1)
ν_{47}	a″	2857.7	(5.1)	2809.1	(4.7)	2888.3	(5.1)	2839.7	(4.5)
ν_{48}	a″	1150.3	(0.0)	1136.1	(0.1)	1164.9	(0.1)	1153.1	(0.1)
ν_{49}	a″	1006.4	(0.0)	1014.8	(0.0)	1014.6	(0.0)	1014.4	(0.4)
ν_{50}	a″	1004.7	(0.2)	1008.6	(0.1)	1005.6	(0.3)	1016.8	(0.5)
ν_{51}	a″	997.6	(0.0)	997.8	(0.2)	994.1	(1.1)	990.9	(0.2)
ν_{52}	a″	969.7	(2.2)	972.8	(1.4)	983.5	(2.0)	983.5	(2.5)
ν_{53}	a″	936.8	(21.8)	950.3	(1.0)	973.1	(0.1)	976.6	(0.1)
ν_{54}	a″	880.7	(19.8)	884.7	(2.4)	882.0	(10.6)	900.2	(0.1)
ν_{55}	a″	857.1	(26.3)	919.8	(42.5)	875.4	(22.2)	878.8	(28.5)
ν_{56}	a″	803.3	(0.1)	791.2	(0.4)	810.3	(29.9)	796.8	(23.8)
ν_{57}	a″	777.1	(0.1)	835.3	(2.7)	766.0	(13.3)	835.1	(9.9)
ν_{58}	a″	761.1	(47.6)	757.0	(26.3)	743.5	(13.9)	743.1	(15.2)
ν_{59}	a″	729.3	(0.6)	733.2	(9.1)	714.7	(48.1)	712.2	(39.7)
ν_{60}	a″	639.1	(21.0)	635.9	(28.2)	626.2	(0.7)	627.4	(0.5)
ν_{61}	a″	514.3	(3.5)	502.5	(1.2)	520.3	(2.9)	516.5	(2.4)
ν_{62}	a″	491.3	(4.2)	478.7	(5.5)	488.6	(1.7)	484.4	(1.5)
ν_{63}	a″	407.2	(3.0)	400.6	(6.0)	432.4	(0.7)	427.7	(0.6)
ν_{64}	a″	395.8	(5.2)	375.2	(2.5)	398.3	(3.9)	392.8	(5.2)
ν_{65}	a″	282.8	(0.0)	283.3	(0.8)	275.5	(7.5)	275.3	(7.9)
ν_{66}	a″	227.3	(0.0)	208.9	(0.0)	217.9	(2.0)	214.6	(2.3)
ν_{67}	a″	186.5	(8.2)	181.9	(7.7)	173.8	(6.3)	178.9	(5.9)
ν_{68}	a″	99.2	(1.0)	91.8	(1.4)	104.3	(0.8)	100.8	(0.9)
ν_{69}	a″	67.6	(0.8)	61.4	(0.7)	85.5	(0.1)	75.5	(0.2)
			overtone/combination mode[b]				overtone/combination mode[b]		
			$2\nu_{40}$	1395.6	(17.7)		$2\nu_{40}$	1374.7	(15.7)
			$\nu_{40} + \nu_{37}$	1564.3	(17.4)		$\nu_{42} + \nu_{36}$	1495.9	(42.0)
			$\nu_{40} + \nu_{38}$	1519.9	(33.2)		$\nu_{45} + \nu_{32}$	1530.7	(21.6)
			$\nu_{40} + \nu_{39}$	1409.2	(98.2)		$\nu_{46} + \nu_{21}$	1617.0	(24.5)
			$\nu_{41} + \nu_{34}$	1643.5	(16.2)		$\nu_{58} + \nu_{54}$	1675.2	(19.2)
			$\nu_{41} + \nu_{35}$	1601.2	(19.9)		$\nu_{59} + \nu_{57}$	1504.2	(71.7)

$\nu_{41} + \nu_{36}$	1526.8	(90.6)	$\nu_{60} + \nu_{57}$	1419.1	(44.1)
$\nu_{41} + \nu_{37}$	1472.3	(44.8)	$\nu_{61} + \nu_{56}$	1329.1	(73.8)
$\nu_{42} + \nu_{33}$	1594.2	(51.7)	$\nu_{62} + \nu_{51}$	1477.6	(40.8)
$\nu_{43} + \nu_{32}$	1615.6	(15.5)	$\nu_{62} + \nu_{53}$	1461.1	(34.6)
$\nu_{43} + \nu_{36}$	1416.8	(123.8)	$\nu_{63} + \nu_{53}$	1404.2	(22.2)
$\nu_{44} + \nu_{36}$	1354.3	(26.1)	$\nu_{64} + \nu_{51}$	1382.3	(36.9)
$\nu_{45} + \nu_{27}$	1635.3	(26.1)			
$\nu_{45} + \nu_{29}$	1585.9	(25.1)			
$\nu_{45} + \nu_{31}$	1565.0	(20.0)			
$\nu_{45} + \nu_{50}$	1517.8	(49.4)			
$\nu_{46} + \nu_{30}$	1409.5	(196.2)			
$\nu_{58} + \nu_{54}$	1666.5	(52.5)			
$\nu_{60} + \nu_{53}$	1579.9	(38.3)			
$\nu_{60} + \nu_{58}$	1417.1	(16.2)			
$\nu_{61} + \nu_{49}$	1514.3	(46.5)			
$\nu_{61} + \nu_{50}$	1508.6	(62.7)			
$\nu_{61} + \nu_{55}$	1386.7	(16.2)			
$\nu_{62} + \nu_{48}$	1618.3	(70.9)			
$\nu_{62} + \nu_{55}$	1371.1	(108.8)			
$\nu_{62} + \nu_{59}$	1236.2	(52.5)			
$\nu_{63} + \nu_{51}$	1395.4	(106.4)			
$\nu_{63} + \nu_{53}$	1339.0	(29.0)			
$\nu_{67} + \nu_{40}$	883.5	(22.5)			

[a]Harmonic vibrational wavenumbers are scaled according to $0.9548x + 27.9$ for wavenumbers > 2000 cm^{-1} and $0.9804x + 2.33$ for wavenumbers < 2000 cm^{-1}. Harmonic IR intensities (in km mol^{-1}) are listed in parentheses. [b]Overtone and combination wavenumbers with the intensity > 15 km mol^{-1} were listed.

Table S3. Scaled Harmonic Vibrational Wavenumbers and IR Intensities of 4a- and 8a-$H^+C_{14}H_{10}$ Predicted with the B3LYP/6-311++G(d,p) Method

mode	sym.	4a-$H^+C_{14}H_{10}$		8a-$H^+C_{14}H_{10}$	
		calculation[a]		calculation[a]	
ν_1	a″	3100.1	(0.0)	3103.5	(0.4)
ν_2	a″	3090.2	(0.4)	3092.1	(0.4)
ν_3	a″	3085.3	(1.4)	3088.9	(0.6)
ν_4	a″	3081.5	(0.3)	3083.5	(0.6)
ν_5	a″	3077.6	(0.1)	3079.9	(0.2)
ν_6	a″	3076.0	(0.3)	3076.2	(0.3)
ν_7	a″	3071.9	(0.2)	3070.7	(0.3)
ν_8	a″	3066.6	(0.4)	3067.4	(0.4)
ν_9	a″	3066.4	(0.3)	3066.4	(0.7)
ν_{10}	a″	3059.9	(0.5)	3063.3	(0.2)
ν_{11}	a″	2689.2	(27.9)	2662.0	(42.8)
ν_{12}	a″	1624.4	(50.7)	1636.9	(28.0)
ν_{13}	a″	1609.1	(71.4)	1623.1	(49.4)
ν_{14}	a″	1584.7	(99.8)	1606.0	(79.2)
ν_{15}	a″	1558.7	(37.4)	1550.6	(6.9)
ν_{16}	a″	1519.9	(74.3)	1524.8	(26.0)
ν_{17}	a″	1503.4	(60.5)	1475.5	(33.3)
ν_{18}	a″	1457.7	(29.5)	1461.6	(151.9)
ν_{19}	a″	1438.5	(86.8)	1450.4	(314.5)
ν_{20}	a″	1430.9	(359.8)	1414.8	(18.0)
ν_{21}	a″	1405.1	(52.6)	1405.5	(15.4)
ν_{22}	a″	1358.8	(14.9)	1354.8	(49.2)
ν_{23}	a″	1334.9	(89.3)	1349.0	(56.8)
ν_{24}	a″	1289.6	(1.6)	1308.6	(100.7)
ν_{25}	a″	1263.6	(29.4)	1270.5	(11.0)
ν_{26}	a″	1237.7	(26.7)	1236.6	(32.5)
ν_{27}	a″	1222.5	(21.7)	1217.3	(37.6)
ν_{28}	a″	1205.2	(52.7)	1182.8	(3.3)
ν_{29}	a″	1180.0	(4.0)	1179.9	(28.3)
ν_{30}	a″	1174.4	(64.7)	1173.4	(3.7)
ν_{31}	a″	1158.0	(15.2)	1152.9	(7.6)
ν_{32}	a″	1146.9	(6.4)	1136.5	(7.0)
ν_{33}	a″	1092.1	(0.8)	1089.6	(2.0)
ν_{34}	a″	1058.1	(25.3)	1042.9	(1.9)

ν_{35}	a″	1039.4	(13.8)	1027.2	(2.0)
ν_{36}	a″	1026.6	(0.9)	1015.3	(1.4)
ν_{37}	a″	1017.0	(3.1)	1012.9	(13.4)
ν_{38}	a″	1007.1	(0.2)	1008.9	(1.1)
ν_{39}	a″	1002.2	(0.8)	1001.9	(3.3)
ν_{40}	a″	1001.3	(6.9)	981.5	(1.6)
ν_{41}	a″	971.1	(5.3)	975.3	(1.0)
ν_{42}	a″	967.2	(5.3)	945.9	(35.5)
ν_{43}	a″	952.6	(7.5)	944.9	(26.1)
ν_{44}	a″	893.2	(33.7)	883.2	(3.4)
ν_{45}	a″	878.0	(7.5)	881.2	(6.9)
ν_{46}	a″	845.8	(0.4)	848.1	(2.3)
ν_{47}	a″	836.4	(28)	805.8	(89.0)
ν_{48}	a″	814.3	(2.9)	805.2	(5.2)
ν_{49}	a″	793.5	(6.5)	774.6	(7.2)
ν_{50}	a″	759.7	(32.0)	767.9	(8.9)
ν_{51}	a″	723.4	(54.6)	720.2	(44.9)
ν_{52}	a″	708.9	(6.1)	696.2	(10.6)
ν_{53}	a″	687.0	(6.0)	676.2	(28.4)
ν_{54}	a″	670.0	(2.6)	666.3	(8.0)
ν_{55}	a″	599.7	(1.6)	605.2	(0.9)
ν_{56}	a″	539.9	(3.8)	542.6	(4.3)
ν_{57}	a″	531.2	(9.3)	534.3	(1.3)
ν_{58}	a″	506.5	(10.2)	511.8	(1.4)
ν_{59}	a″	486.2	(4.1)	487.4	(1.9)
ν_{60}	a″	463.7	(6.9)	450.8	(1.5)
ν_{61}	a″	415.0	(0.9)	434.9	(7.0)
ν_{62}	a″	410.4	(5.5)	406.3	(5.3)
ν_{63}	a″	390.1	(0.9)	386.3	(5.9)
ν_{64}	a″	367.2	(3.4)	353.2	(1.8)
ν_{65}	a″	237.6	(1.5)	247.7	(2.2)
ν_{66}	a″	215.9	(0.0)	209.6	(0.6)
ν_{67}	a″	204.2	(3.5)	194.1	(3.5)
ν_{68}	a″	93.3	(0.9)	93.5	(0.3)
ν_{69}	a″	77.5	(0.1)	86.3	(1.0)

[a]Harmonic vibrational wavenumbers are scaled according to $0.9548x + 27.9$ for wavenumbers > 2000 cm^{-1} and $0.9804x + 2.3$ for wavenumbers < 2000 cm^{-1}. Harmonic IR intensities (in km mol^{-1}) are listed in parentheses.

Table S4. Comparison of Observed Vibrational Wavenumbers of $C_{14}H_{10}$ in Solid *p*-H_2 and Ar with Predicted Scaled Harmonic Vibrational Wavenumbers and IR Intensities of $C_{14}H_{10}$ with Various Methods

calculation				experiment				
B3LYP/6-311G++(d,p) [a]		B3LYP/6-31G* [b]		*p*-H_2 matrix		Ar matrix [b]		gas-phase [c]
/ cm^{-1}	km mol^{-1}	/ cm^{-1}	km mol^{-1}	/ cm^{-1}		/ cm^{-1}		/ cm^{-1}
3087.4	(19.5)	3093.3	(32.9)	3108.6	(1.0) [d]	3106.0		3102.0
3075.6	(14.5)	3082.5	(36.3)	3082.5	(3.5)	3078.2		3082.0
3069.1	(0.8)	3075.2	(2.7)					
3066.1	(37.2)	3070.7	(52.8)	3072.2	(3.5)	3070.9		3068.0
3059.7	(32.2)	3063.6	(47.1)	3065.3	(6.3)	3063.6		3061.0
3054.1	(13.8)	3057.0	(18.3)	3058.1	(3.0)	3053.9		
3052.9	(0.0)							
3044.4	(1.9)	3045.8	(2.9)					
3043.7	(3.1)	3045.1	(5.5)	3048.1	(2.0)	3046.4		
3040.7	(0.1)							
				3036.3	(2.0)	3036.9		
				3032.6	(3.3)	3031.1		3032.0
				3016.3	(0.8)	3013.7		3015.0
				2968.9	(0.5)			
				2961.9	(0.5)	2951.0		
						1946.7	(4.0) [d]	
1627.5	(0.2)							
1622.7	(0.1)	1610.5	(0.8)			1602.8	(0.4)	1608.0
1610.5	(1.9)	1595.1	(4.5)			1597.9	(1.0)	
1576.8	(0.1)							
1532.0	(1.9)	1521.6	(1.8)	1530.7	(2.8)	1531.8	(2.0)	1529.5
						1530.1		
1505.6	(5.9)	1497.3	(7.1)	1504.6	(8.5)	1505.9	(9.0)	1503.8
						1504.7		
1462.2	(10.6)	1461.7	(14.3)	1460.2	(16.3)	1460.4	(14.0)	1458.0
1443.1	(3.6)	1443.9	(3.6)	1447.7	(2.0)	1447.9	(0.5)	1446.0

						1436.2	(0.7)	
						1430.7	(2.0)	1431.0
						1427.5	(1.0)	
1423.3	(1.2)							
1418.3	(0.4)	1417.8	(1.0)			1419.0	(1.0)	
1349.6	(0.8)	1343.4	(2.2)			1351.4	(1.0)	1358.0
1343.3	(0.1)							1340.0
1300.3	(2.6)	1298.8	(1.6)	1302.7	(3.3)	1302.9	(4.0)	1299.0
1280.8	(0.0)							1270.0
1243.1	(10.2)	1250.3	(9.4)	1245.5	(6.8)	1250.6	(11.0)	1244.0
						1245.8		
1219.7	(0.5)	1224.6	(0.9)	1221.3	(1.0)	1223.8	(0.6)	1236.0
1202.4	(1.8)	1203.0	(2.0)	1201.5	(1.3)	1202.7	(3.0)	1200.0
1167.9	(0.5)							
1164.8	(0.2)			1165.0	(0.8)	1165.4	(2.0)	1163.0
1152.6	(0.0)							
1144.4	(2.2)	1148.3	(1.3)	1143.2	(2.5)	1144.1	(3.0)	1142.0
1094.8	(2.3)	1093.0	(0.9)	1094.8	(2.3)	1095.9	(2.0)	1093.8
						1094.5		
1042.0	(1.7)			1040.7	(5.5)	1044.3	(6.0)	1040.0
1041.6	(7.2)	1038.3	(3.6)	1039.9	(6.8)	1039.9		
998.9	(2.5)	999.0	(1.4)	1002.7	(5.3)	1003.7	(2.0)	1002.1
						1002.5		
974.5	(0.0)							
974.4	(0.0)							
964.3	(0.0)							
949.7	(3.3)	950.3	(4.2)	949.3	(4.5)	948.2	(3.0)	948.1
940.0	(0.0)							
872.6	(2.1)	870.2	(1.4)	876.7	(2.0)	877.6	(2.0)	875.8
864.6	(11.9)	871.4	(11.1)	865.5	(17.1)	864.9	(12.0)	862.5
857.2	(0.0)							
828.6	(0.1)			831.5	(0.3)	833.0	(2.0)	

813.3	(59.9)	817.0	(59.7)	813.2	(81.4)	812.8	(69.0)	809.6
785.3	(0.0)							
754.2	(0.0)							
734.7	(90.2)	736.6	(74.6)	735.9	(100.0)	735.0	(100.0)	732.3
714.8	(2.1)	716.7	(2.4)					
713.5	(1.6)	715.1	(1.5)			714.7	(4.0)	708.5
						710.6		
710.9	(0.1)							
621.7	(5.3)	627.8	(4.6)	617.7	(7.3)	618.8	(6.0)	618.0
						617.5		
589.8	(0.0)							
548.2	(0.3)							
535.4	(0.0)	500.1	(0.8)					502.3
499.8	(5.6)	498.4	(3.9)	493.5	(6.3)	494.0	(4.0)	493.5
498.4	(1.0)							
439.7	(1.9)							
430.6	(7.9)							431.0
								424.0
405.5	(0.5)							
395.5	(0.0)							398.0
244.5	(0.5)							
239.5	(0.0)							
224.8	(4.1)							225.0
100.5	(0.9)							
95.5	(0.0)							

[a]Harmonic vibrational wavenumbers scaled according to $0.9548x + 27.9$ for wavenumbers > 2000 cm^{-1} and $0.9804x + 2.3$ for wavenumbers < 2000 cm^{-1}. Harmonic IR intensities (in km mol^{-1}) are listed in parentheses. [b]From references 1 and 2; the scaling factor is 0.98. [c]From reference 3. [d]Percentage integrated intensities are relative to the most intense band.

Table S5. Summary of Estimated Mixing Ratios of Each Species in the Electron-bombarded $C_{14}H_{10}$/*p*-H_2 Matrix Experiment

group	assignment	mixing ratio	integration range (cm^{-1})
parent	$C_{14}H_{10}$	31.8 ± 1.7 ppm	620−616, 740−733, 816−812, 867−864, 951−947, 1004−1001, 1043−1038, 1248−1241, 1463−1456, and 1507−1501
W	9-$H^+C_{14}H_{10}$	132 ± 27 ppb	1487−1484, 1455−1453, and 1259−1256
X	1-$H^+C_{14}H_{10}$	78 ± 14 ppb	1581−1578, 1392−1388, and 1353−1349
Y	3-$H^+C_{14}H_{10}$	128 ± 42 ppb	1589−1585 and 1346−1342
Z	4-$H^+C_{14}H_{10}$	120 ± 37 ppb	1625−1622, 1481−1476, and 1407−1396

Table S6. Vertical Excitation Wavelengths and Oscillator Strengths of Electronic Excitation of $C_{14}H_{10}$ and 9-, 1-, 2-, 3-, 4-, 4a-, and 8a-$H^+C_{14}H_{10}$ Predicted with the TD-B3LYP/6-311++G(d,p) Method

#	$C_{14}H_{10}$					9-$H^+C_{14}H_{10}$				
	λ[a]	$f/10^{-2}$ [b]	assignment			λ[a]	$f/10^{-2}$ [b]	assignment		
1	314	0.21	HOMO	→	LUMO+1	527	5.98	HOMO	→	LUMO
2	296	6.06	HOMO	→	LUMO	478	9.20	HOMO–1	→	LUMO
3	265	10.34	HOMO−1	→	LUMO	320	0.22	HOMO–2	→	LUMO
4	254	61.94	HOMO−1	→	LUMO+1	285	5.74	HOMO–3	→	LUMO
5	246	2.54	HOMO	→	LUMO+2	273	0.01	HOMO–4	→	LUMO
6	243	9.88	HOMO−2	→	LUMO+1	260	4.01	HOMO–1	→	LUMO+1
7	237	0.49	HOMO	→	LUMO+3	260	0.00	HOMO–5	→	LUMO
8	227	0.16	HOMO	→	LUMO+5	247	42.14	HOMO	→	LUMO+1
9	226	0.00	HOMO	→	LUMO+4	227	0.05	HOMO–7	→	LUMO
10	223	0.00	HOMO−1	→	LUMO+3	225	24.07	HOMO	→	LUMO+2
11	219	0.33	HOMO	→	LUMO+6	220	2.64	HOMO–6	→	LUMO
12	217	13.20	HOMO−2	→	LUMO	219	0.03	HOMO–8	→	LUMO

#	1-$H^+C_{14}H_{10}$					2-$H^+C_{14}H_{10}$				
	λ[a]	$f/10^{-2}$ [b]	assignment			λ[a]	$f/10^{-2}$ [b]	assignment		
1	523	14.94	HOMO	→	LUMO	555	3.60	HOMO	→	LUMO
2	471	1.99	HOMO–1	→	LUMO	382	17.67	HOMO–1	→	LUMO
3	316	16.87	HOMO–2	→	LUMO	327	3.44	HOMO–2	→	LUMO
4	288	0.52	HOMO	→	LUMO+1	271	0.00	HOMO–4	→	LUMO
5	269	2.28	HOMO–1	→	LUMO+1	270	49.85	HOMO	→	LUMO+1
6	267	0.00	HOMO–4	→	LUMO	263	3.00	HOMO–3	→	LUMO
7	260	0.08	HOMO–5	→	LUMO	251	7.41	HOMO–1	→	LUMO+1
8	257	1.12	HOMO–3	→	LUMO	245	0.06	HOMO–5	→	LUMO
9	227	14.67	HOMO–6	→	LUMO	237	26.40	HOMO	→	LUMO+2
10	224	0.00	HOMO–7	→	LUMO	228	4.80	HOMO–6	→	LUMO
11	222	53.89	HOMO	→	LUMO+2	224	0.00	HOMO–7	→	LUMO
12	218	0.31	HOMO	→	LUMO+3	222	22.68	HOMO	→	LUMO+3

#	$3\text{-}H^+C_{14}H_{10}$ λ[a]	$f/10^{-2}$ [b]	assignment			$4\text{-}H^+C_{14}H_{10}$ λ[a]	$f/10^{-2}$ [b]	assignment		
1	473	4.13	HOMO–1	→	LUMO	618	2.53	HOMO	→	LUMO
2	462	16.41	HOMO	→	LUMO	421	21.99	HOMO–1	→	LUMO
3	301	2.86	HOMO–2	→	LUMO	333	3.27	HOMO–2	→	LUMO
4	274	1.14	HOMO	→	LUMO+1	282	17.54	HOMO	→	LUMO+1
5	268	6.38	HOMO–3	→	LUMO	277	4.65	HOMO–3	→	LUMO
6	261	0.01	HOMO–4	→	LUMO	277	0.02	HOMO–4	→	LUMO
7	256	0.00	HOMO–5	→	LUMO	265	1.53	HOMO–1	→	LUMO+1
8	248	23.73	HOMO–1	→	LUMO+1	257	0.02	HOMO–5	→	LUMO
9	231	53.76	HOMO	→	LUMO+2	228	0.05	HOMO–7	→	LUMO
10	224	15.54	HOMO–6	→	LUMO	221	31.17	HOMO–6	→	LUMO
11	222	17.26	HOMO	→	LUMO+2	219	2.70	HOMO	→	LUMO+3
12	221	0.02	HOMO–7	→	LUMO	217	0.12	HOMO–8	→	LUMO

#	$4a\text{-}H^+C_{14}H_{10}$ λ[a]	$f/10^{-2}$ [b]	assignment			$8a\text{-}H^+C_{14}H_{10}$ λ[a]	$f/10^{-2}$ [b]	assignment		
1	458	22.58	HOMO	→	LUMO	496	4.71	HOMO	→	LUMO
2	431	0.28	HOMO–1	→	LUMO	401	22.70	HOMO–1	→	LUMO
3	327	2.63	HOMO–2	→	LUMO	297	2.53	HOMO	→	LUMO+1
4	274	21.10	HOMO	→	LUMO+1	286	1.28	HOMO–2	→	LUMO
5	264	3.79	HOMO–1	→	LUMO+1	281	3.43	HOMO–3	→	LUMO
6	257	0.96	HOMO–3	→	LUMO	261	4.44	HOMO–1	→	LUMO+1
7	246	1.75	HOMO–4	→	LUMO	248	11.26	HOMO–4	→	LUMO
8	241	5.44	HOMO–5	→	LUMO	242	7.06	HOMO–1	→	LUMO+2
9	236	5.50	HOMO	→	LUMO+2	238	19.46	HOMO–5	→	LUMO
10	233	3.58	HOMO	→	LUMO+3	232	29.65	HOMO	→	LUMO+2
11	218	1.18	HOMO–6	→	LUMO	218	3.68	HOMO–6	→	LUMO
12	214	1.27	HOMO–1	→	LUMO+2	212	2.56	HOMO–8	→	LUMO

[a]Wavenumber λ in nm. [b]Oscillator strength *f*.

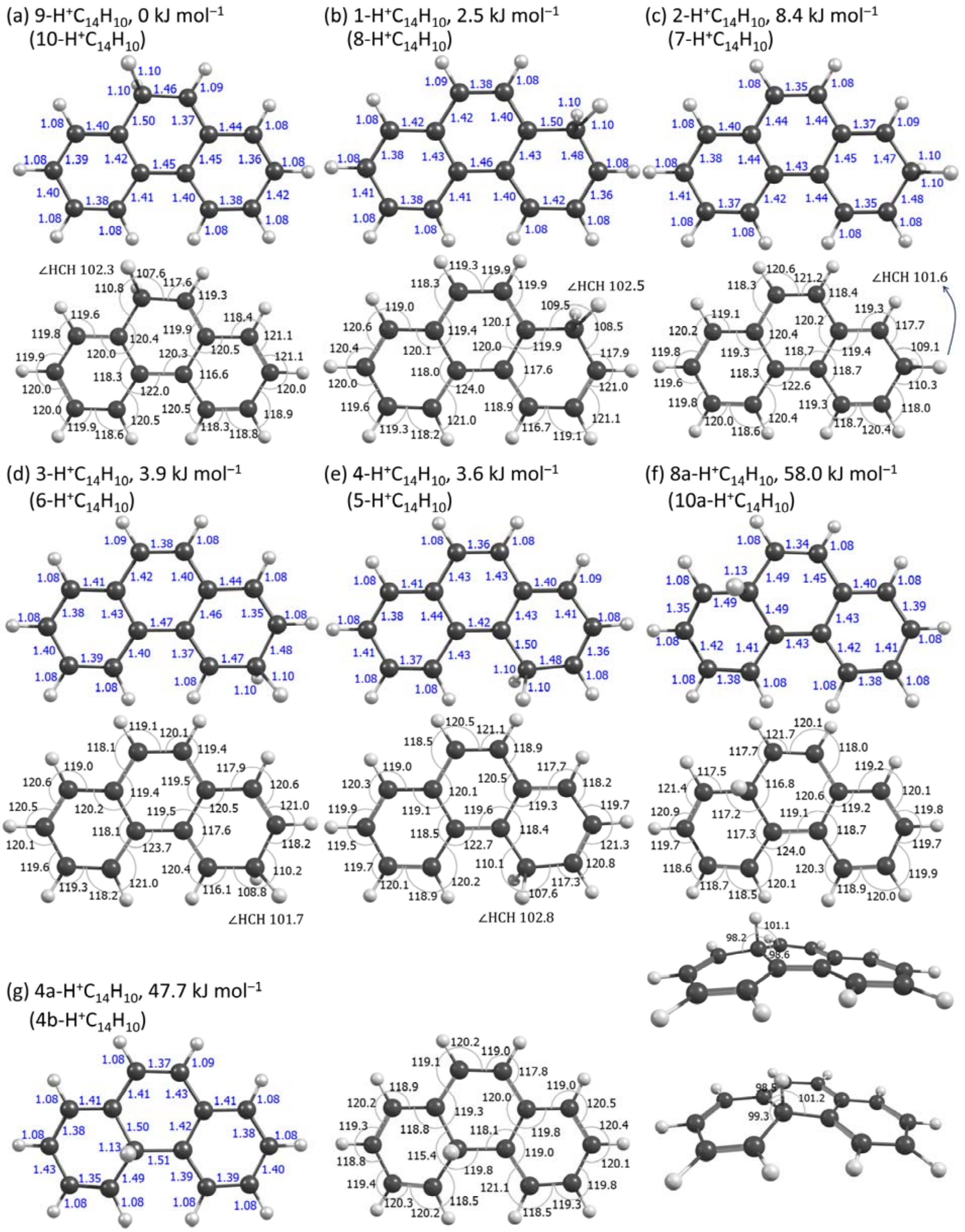

Figure S1. Geometries and relative energies of seven possible isomers of $H^{+}C_{14}H_{10}$. Geometries were optimized with the B3LYP/6-311++G(d,p) method and their energies were calculated with the CCSD(T)/6-311++G(d,p)//B3LYP/6-311++G(d,p) method. Zero-point vibrational energies (ZPVE) were corrected according to harmonic vibrational wavenumbers calculated with the B3LYP/6-311++G(d,p) method. Bond lengths (blue) are in Å and bond angles (black) are in degree.

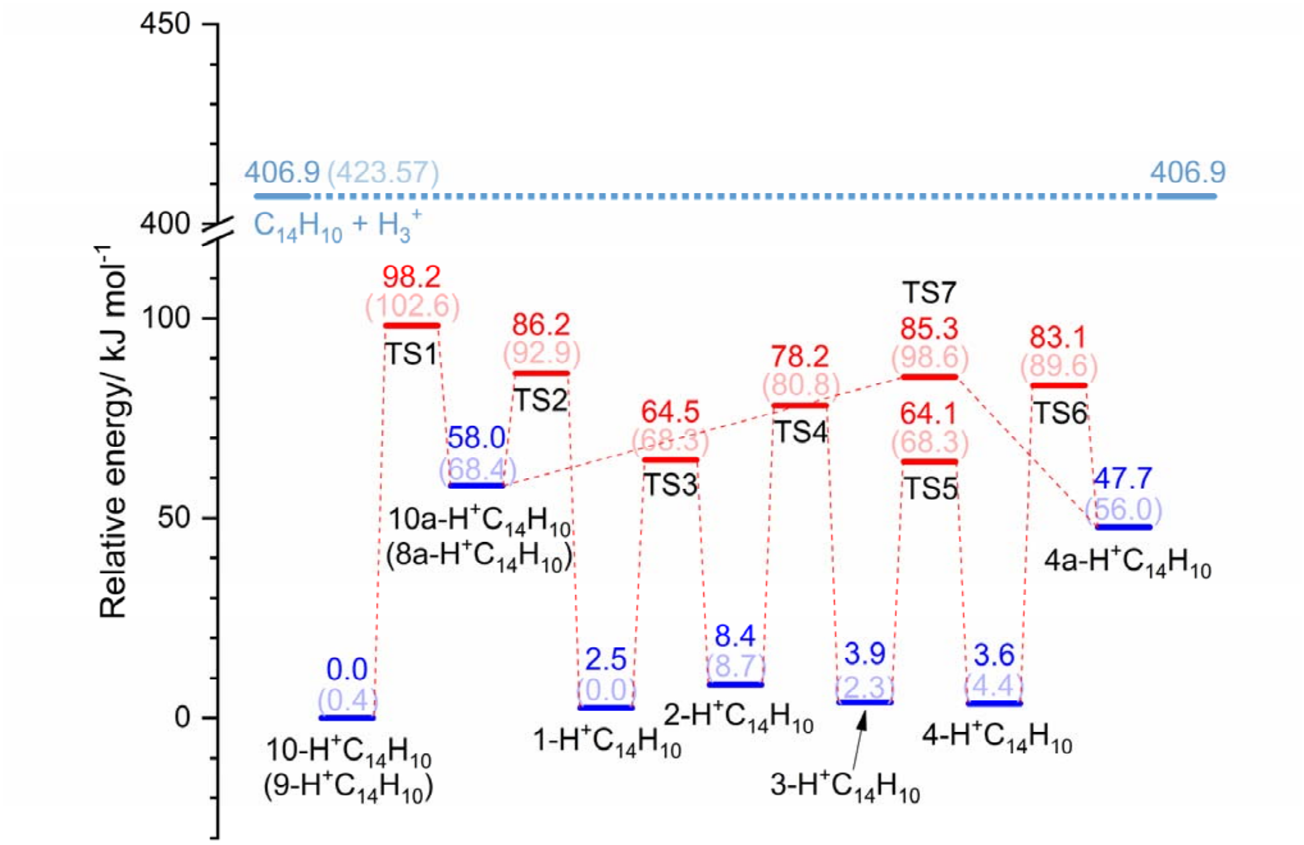

Figure S2. Potential energy scheme for the proton transfer of various isomers of $H^+C_{14}H_{10}$. The energy of all feasible $H^+C_{14}H_{10}$ (+ H_2) is relative to the energy of 9-$H^+C_{14}H_{10}$ (+ H_2). Energies of isomers of $H^+C_{14}H_{10}$, calculated with the CCSD(T)/6-311++G(d,p)//B3LYP/6-311++G(d,p) method and corrected for zero-point vibrational energies (ZPVE) according to harmonic vibrational wavenumbers calculated with the B3LYP/6-311++G(d,p) method, are indicated in blue. Those of transition states for isomerization are in red. ZPVE-corrected B3LYP/6-311G(d,p) energies are listed in the parentheses.

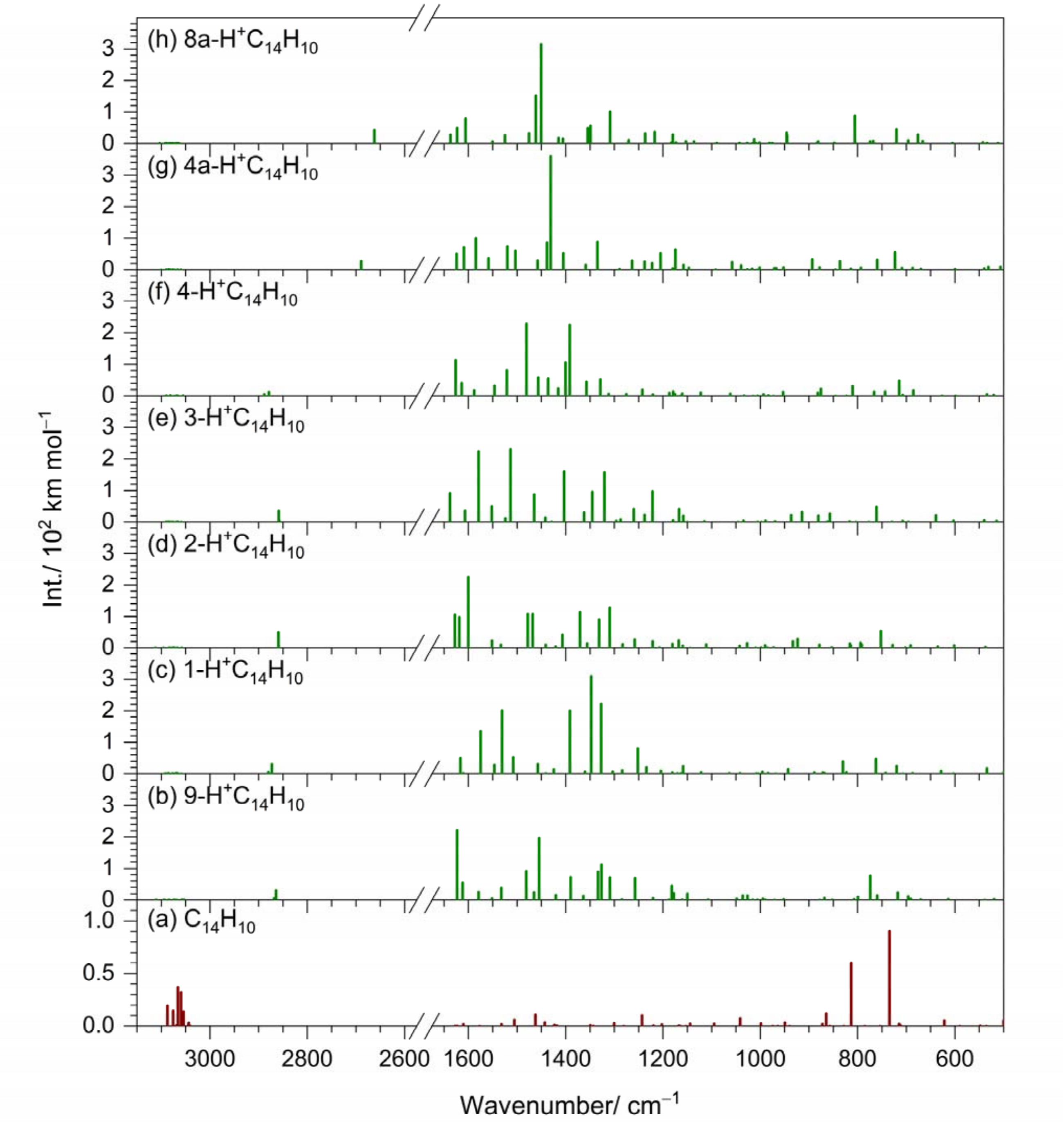

Figure S3. Computed stick spectra of $C_{14}H_{10}$ and isomers of $H^+C_{14}H_{10}$. (a) $C_{14}H_{10}$, (b) 9-$H^+C_{14}H_{10}$, (c) 1-$H^+C_{14}H_{10}$, (d) 2-$H^+C_{14}H_{10}$, (e) 3-$H^+C_{14}H_{10}$, (f) 4-$H^+C_{14}H_{10}$, (g) 4a-$H^+C_{14}H_{10}$, and (h) 8a-$H^+C_{14}H_{10}$. The spectra were based on scaled harmonic vibrational wavenumbers and IR intensities calculated with the B3LYP/6-311++G(d,p) method.

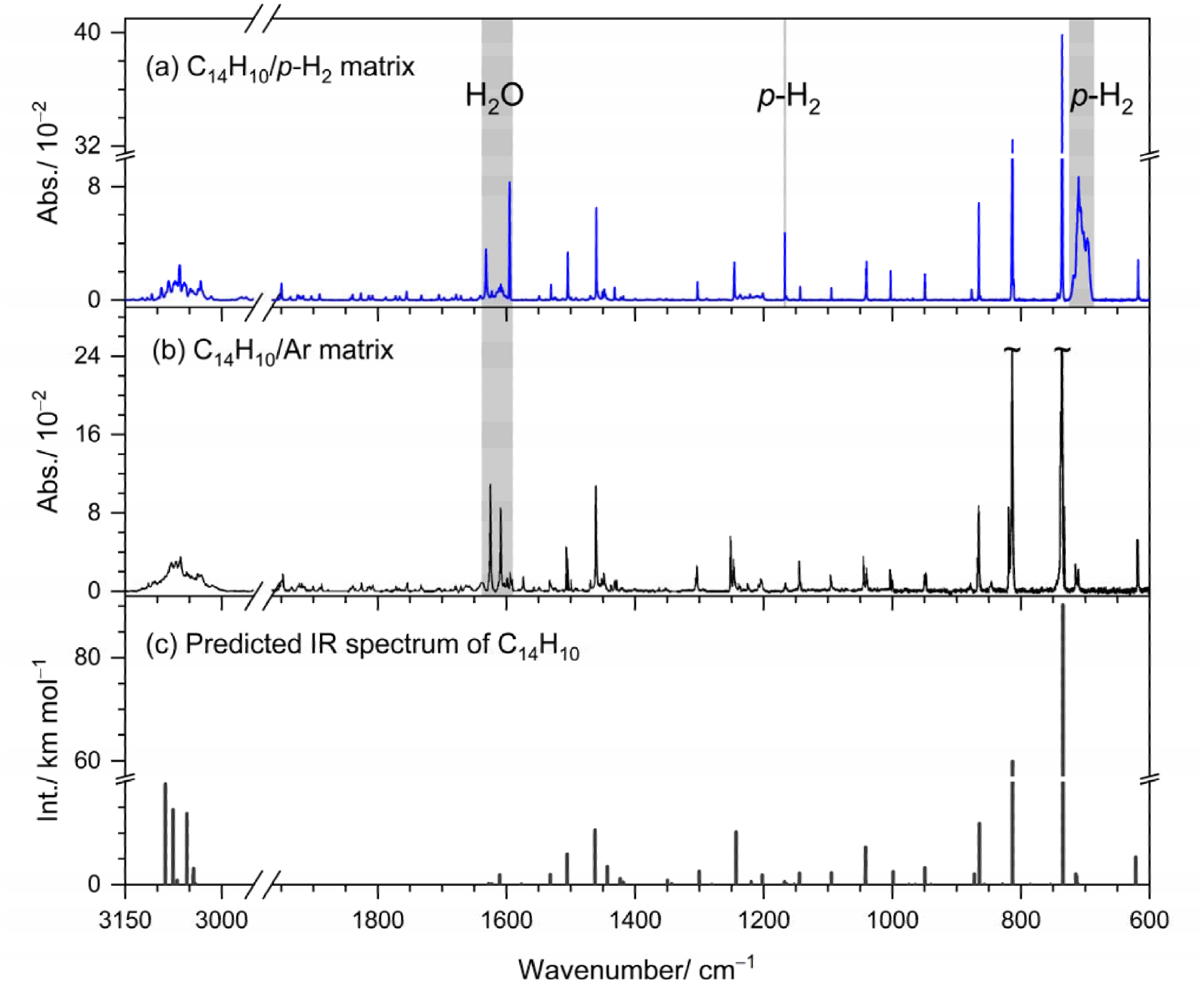

Figure S4. Comparison of the IR absorption spectra of $C_{14}H_{10}$ isolated in solid *p*-H_2 and Ar with the computed stick spectrum of $C_{14}H_{10}$. (a) IR spectrum of $C_{14}H_{10}$ isolated in solid *p*-H_2. Spectral regions subjected to interference from absorption of *p*-H_2 and H_2O are shaded gray. (b) IR spectrum of $C_{14}H_{10}$ isolated in solid Ar. Reproduced from Hudgins and Sandford [1]. Copyright [1998] American Chemical Society. (c) The stick spectrum was simulated according to scaled harmonic vibrational wavenumbers and IR intensities calculated with the B3LYP/6-311++G(d,p) method.

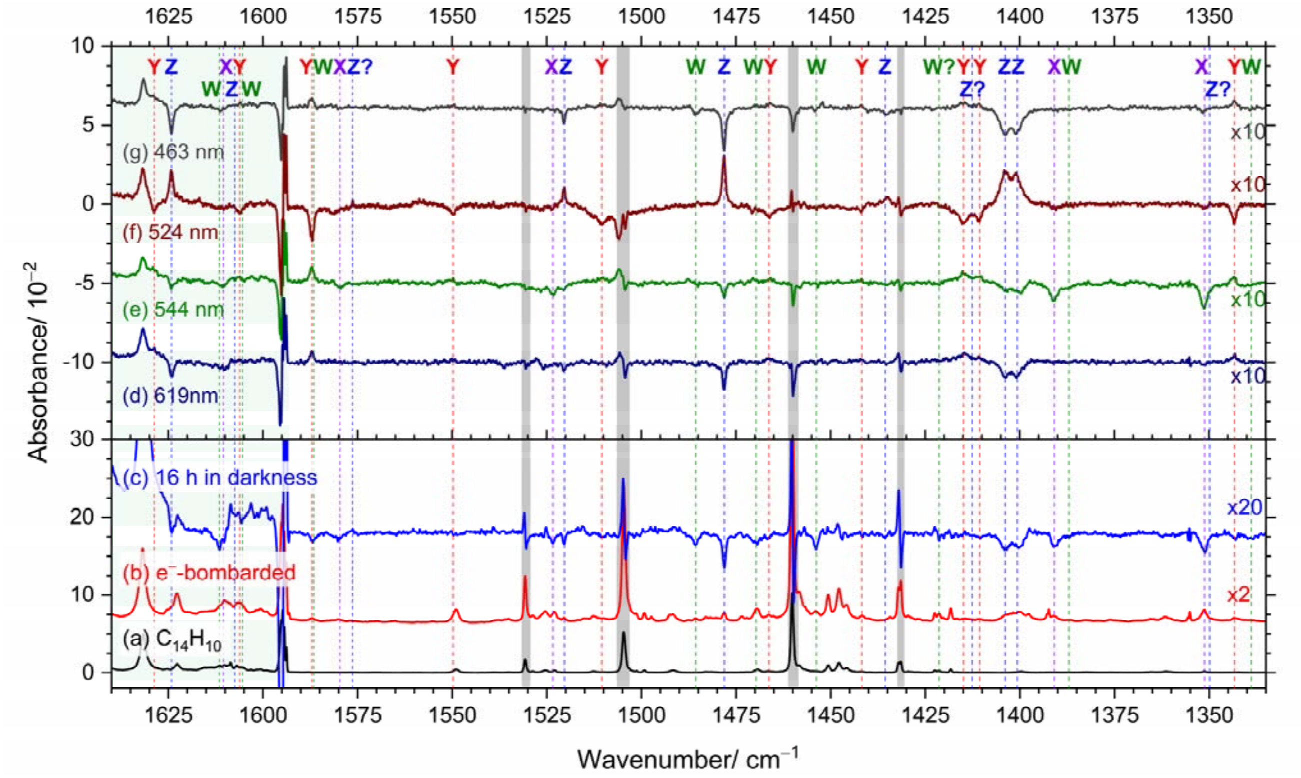

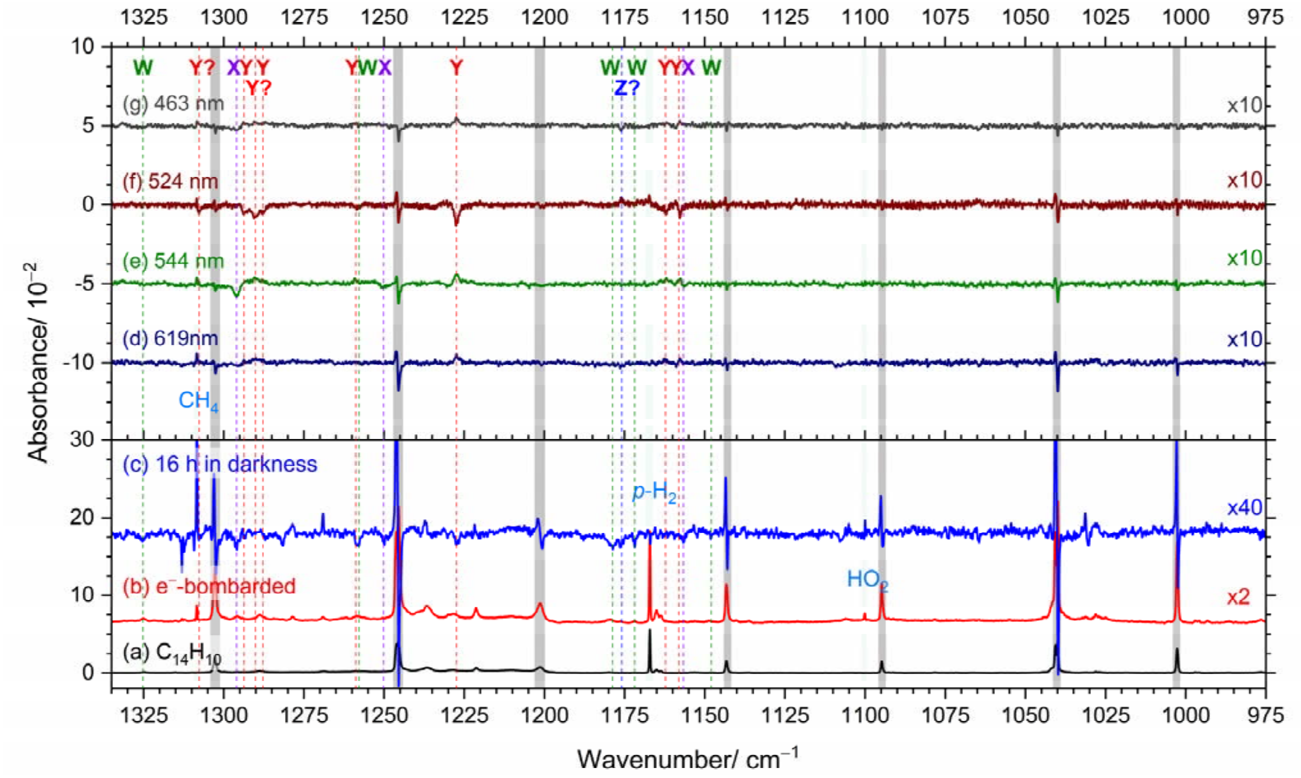

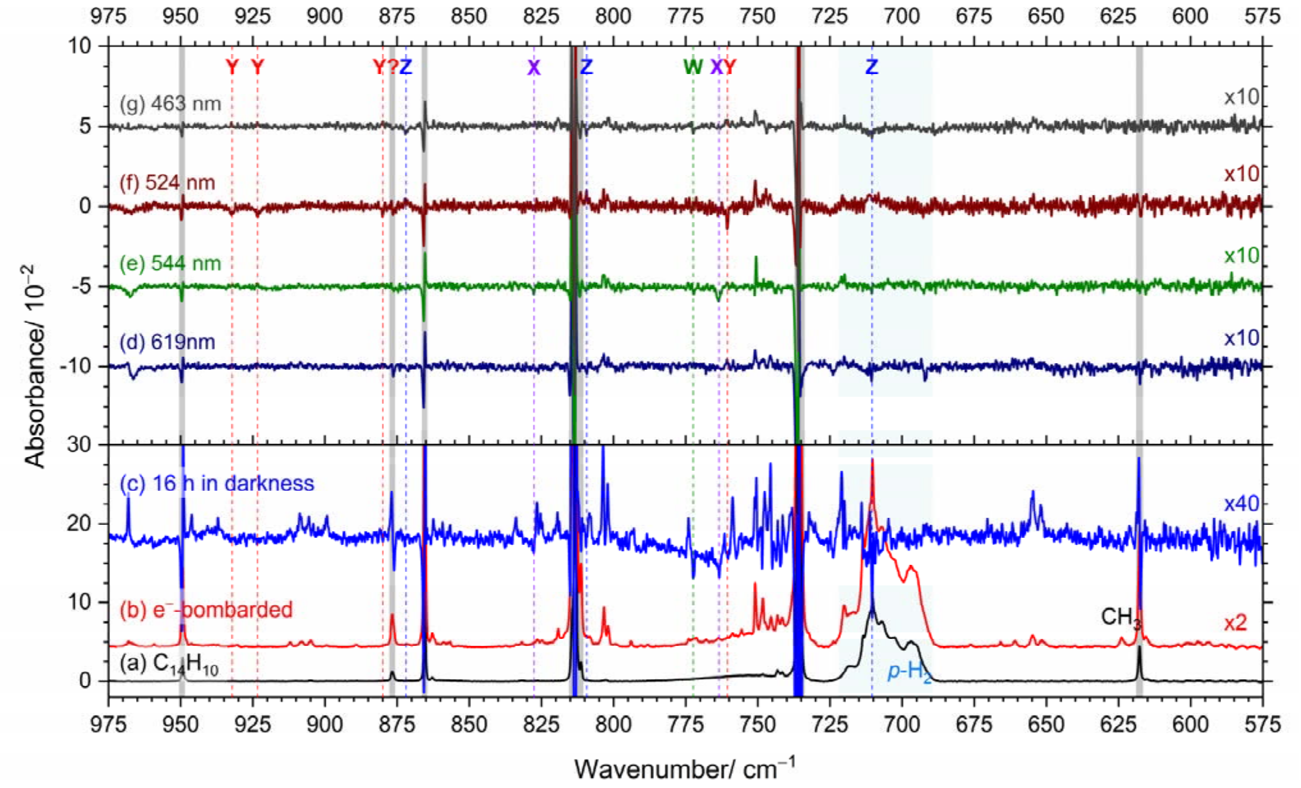

Figure S5. Infrared spectra (in region 975–1640 cm^{-1}) of an electron-bombarded $C_{14}H_{10}$/*p*-H_2 matrix after each experimental step. (a) $C_{14}H_{10}$/*p*-H_2 matrix without electron bombardment. (b) Electron-bombarded $C_{14}H_{10}$/*p*-H_2 matrix. (c) Difference spectrum measured after maintenance of the matrix in darkness for 16 h. in a separate experiment. Difference spectra after secondary irradiation at 619 nm (d), 544 nm (e), 524 nm (f), and 463 nm (g); each irradiation step is 20 min. The lines in groups W, X, Y, and Z are indicated with color-coded labels and dashed lines. Spectral regions subjected to interference from the intense absorption of $C_{14}H_{10}$ are shaded gray. Baselines are shifted for clarity.

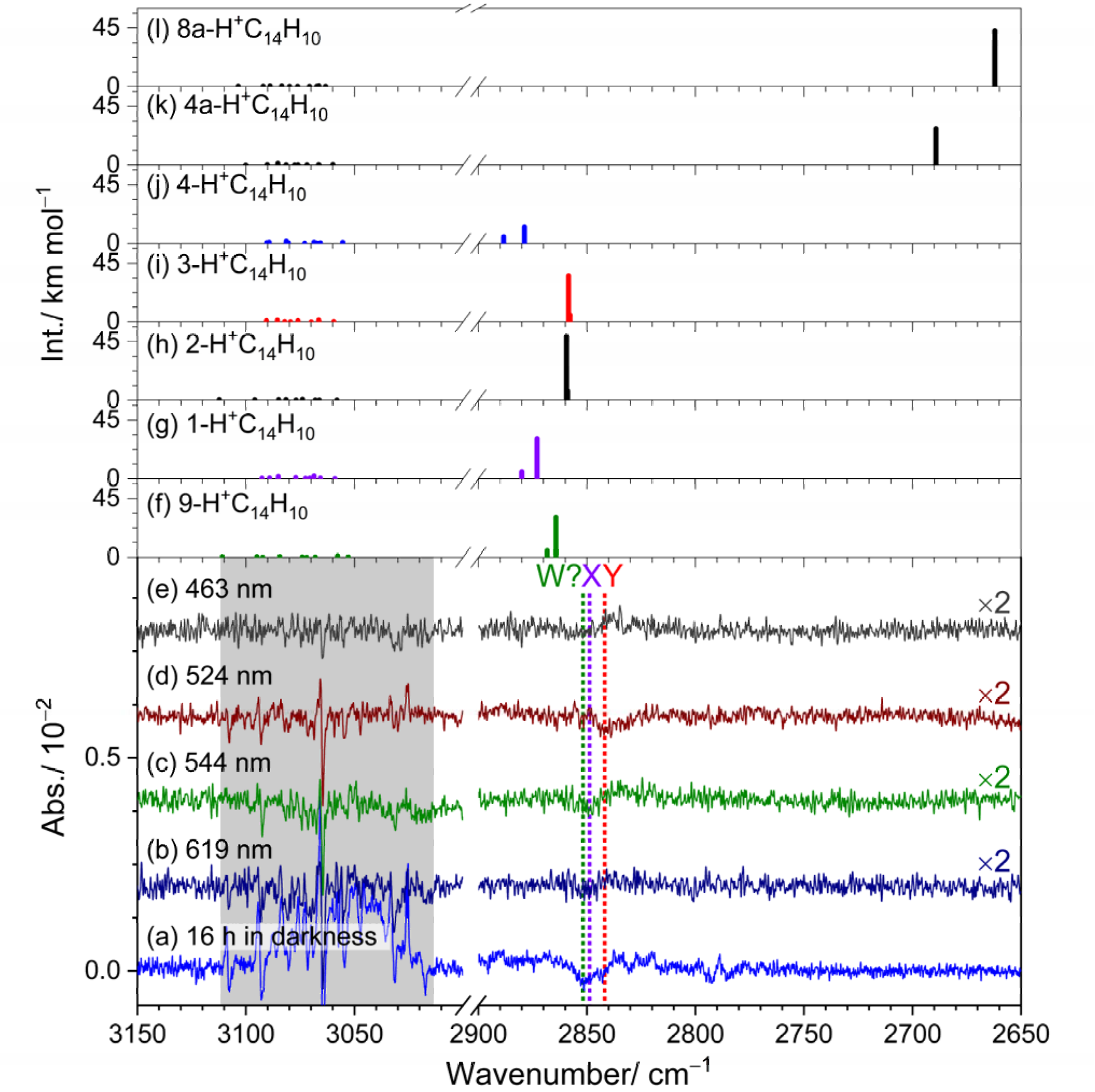

Figure S6. Comparison of observed IR spectra in region 2650–3150 cm^{-1} with stick spectra of seven isomers of $H^+C_{14}H_{10}$. The stick spectra are based on scaled harmonic vibrational wavenumbers and IR intensities calculated with the B3LYP/6-311++G(d,p) method. Difference spectrum measured after maintenance of the matrix in darkness for 16 h (a). Difference spectra, smoothed using the 10-point adjacent-averaging method, after secondary irradiation at 619 nm (b), 544 nm (c), 524 nm (d), and 463 nm (e); each irradiation step is 20 min. Lines in groups W, X, and Y are indicated with color-coded labels and dashed lines. Spectral regions subjected to interference from the intense absorption of $C_{14}H_{10}$ are shaded gray. Baselines are shifted for clarity.

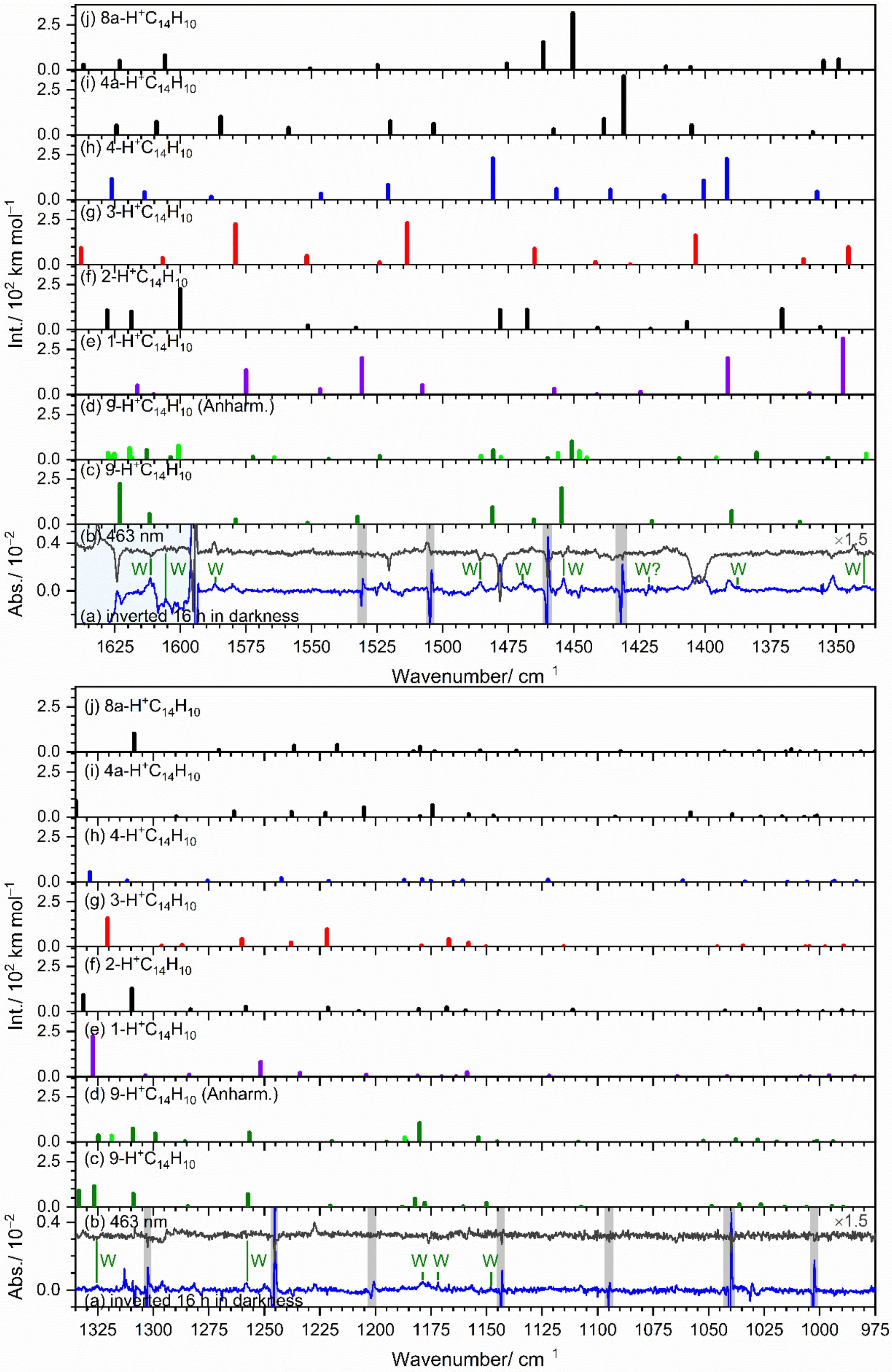

(j) 8a-H$^+$C$_{14}$H$_{10}$
(i) 4a-H$^+$C$_{14}$H$_{10}$
(h) 4-H$^+$C$_{14}$H$_{10}$
(g) 3-H$^+$C$_{14}$H$_{10}$
(f) 2-H$^+$C$_{14}$H$_{10}$
(e) 1-H$^+$C$_{14}$H$_{10}$
(d) 9-H$^+$C$_{14}$H$_{10}$ (Anharm.)
(c) 9-H$^+$C$_{14}$H$_{10}$
(b) 463 nm
(a) inverted 16 h in darkness
×1.5
W
W?
Int./ 10^2 km mol^{-1}
Abs./ 10^{-2}
Wavenumber/ cm^{-1}

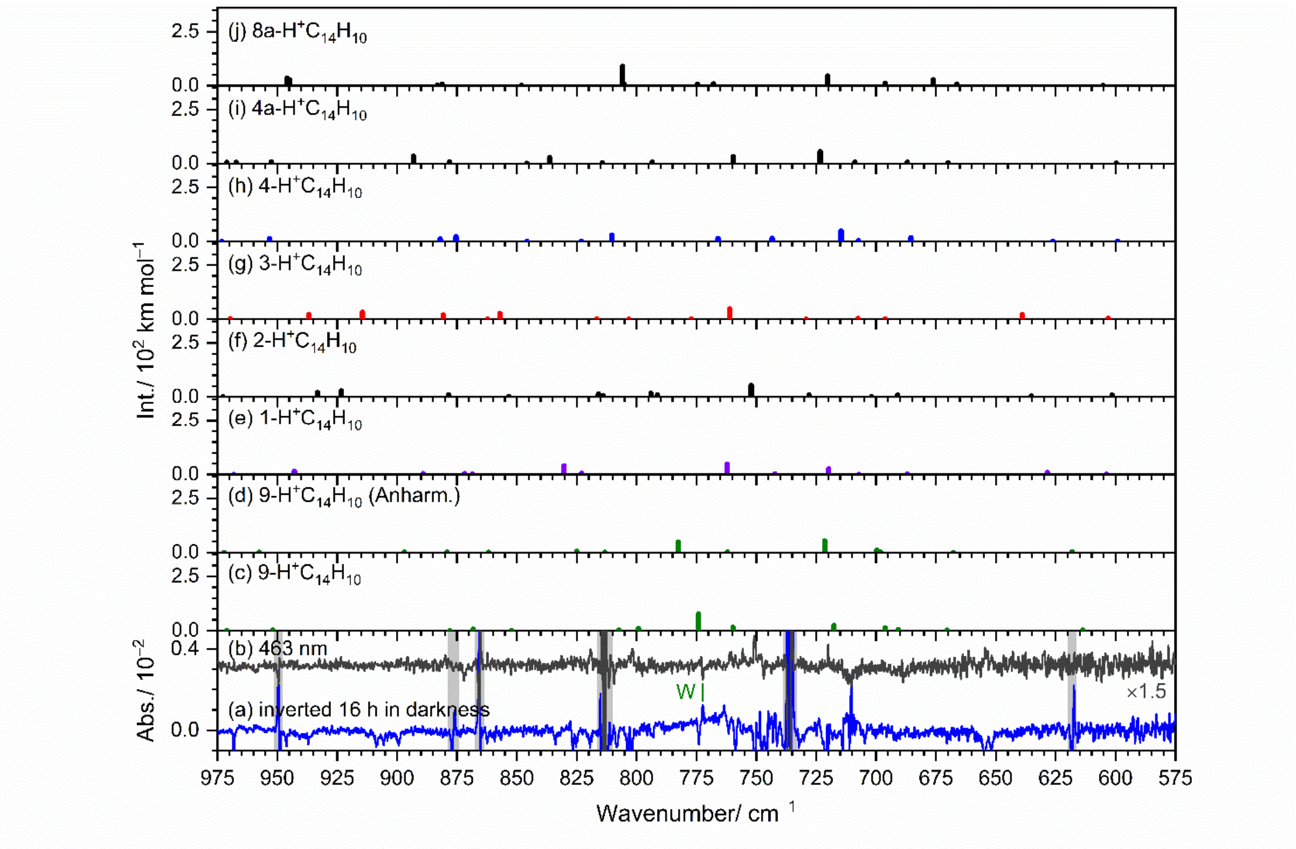

Figure S7. Comparison of lines in group W in region 575−1640 cm^{-1} with stick spectra of seven isomers of $H^+C_{14}H_{10}$. The experimental spectrum (a) after maintenance of the matrix in darkness for 16 h is taken from Figure S5c. The experimental spectrum (b) after irradiation at 463 nm for 20 min is taken from Figure S5g. Lines in group W are indicated with green labels and lines. Spectral regions subjected to interference from the intense absorption of $C_{14}H_{10}$ are shaded gray. Baselines are shifted for clarity. The stick spectra (c) and (e)−(j) are based on scaled harmonic vibrational wavenumbers and IR intensities calculated with the B3LYP/6-311++G(d,p) method. The anharmonic vibrational stick spectrum of 9-$H^+C_{14}H_{10}$ is shown in trace (d), with combination and overtone bands in light color.

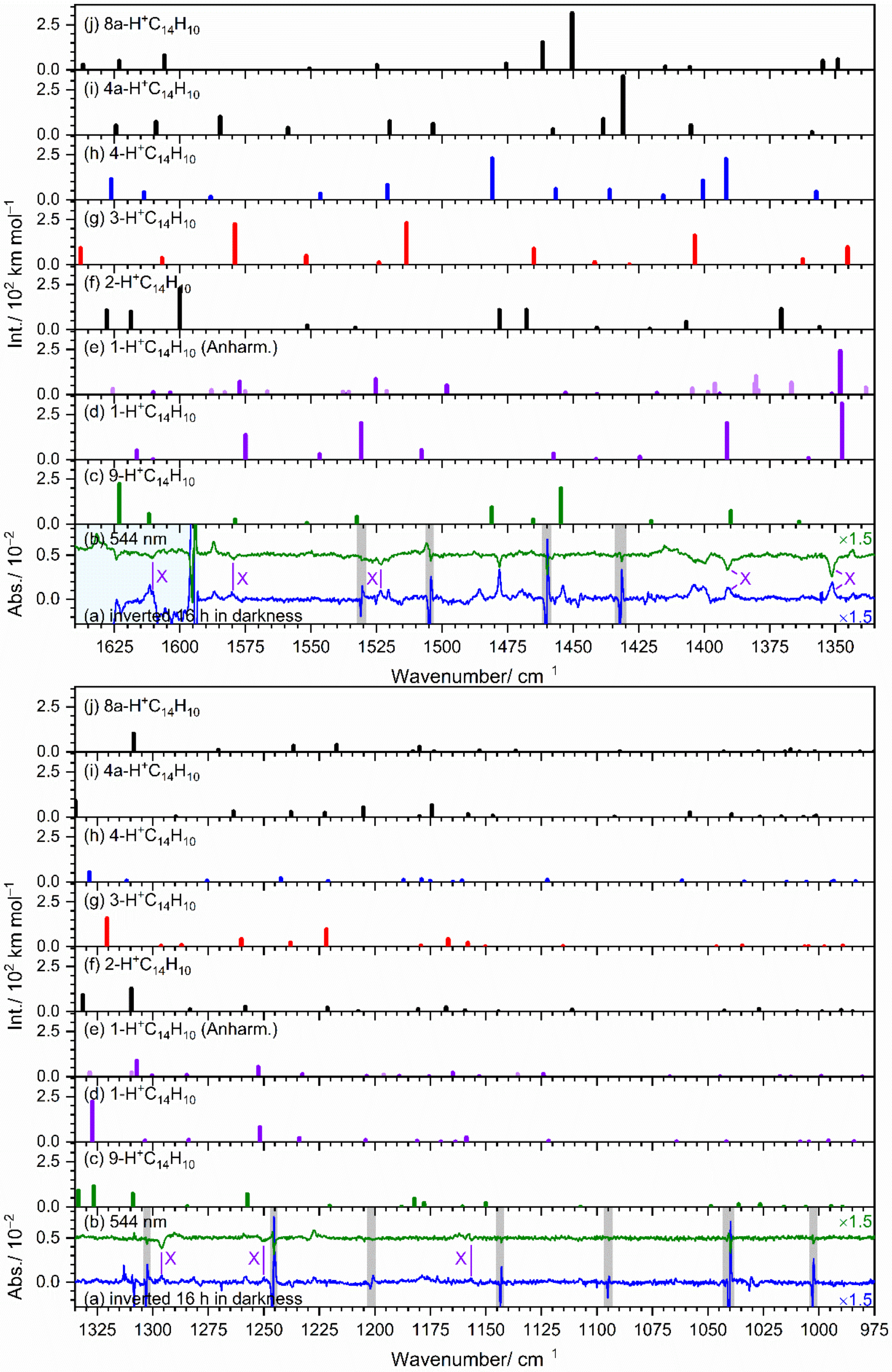

(j) 8a-H+C14H10
(i) 4a-H+C14H10
(h) 4-H+C14H10
(g) 3-H+C14H10
(f) 2-H+C14H10
(e) 1-H+C14H10 (Anharm.)
(d) 1-H+C14H10
(c) 9-H+C14H10
(b) 544 nm
(a) inverted 16 h in darkness
×1.5
×1.5
Int./ 10^2 km mol^-1
Abs./ 10^-2
Wavenumber/ cm-1

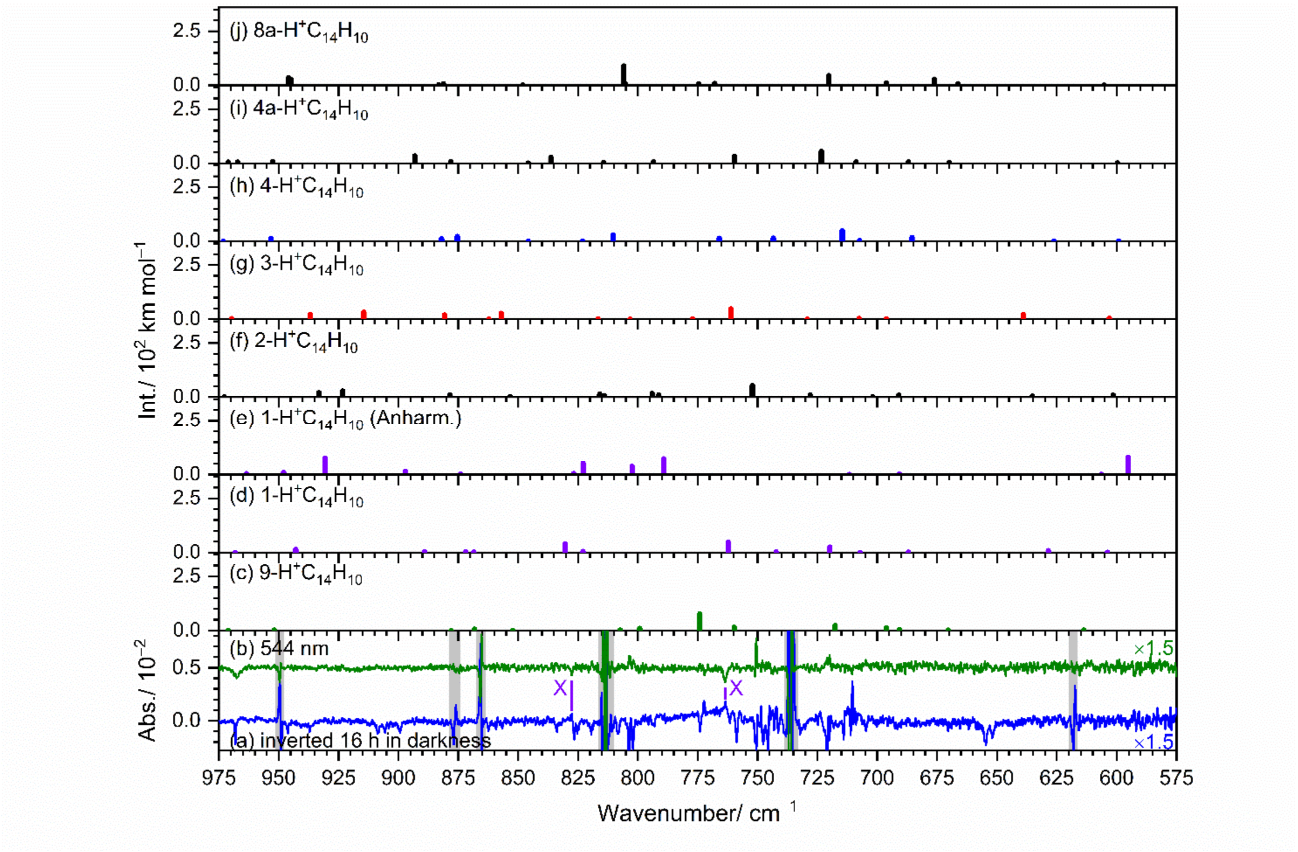

Figure S8. Comparison of lines in group X in region 575−1640 cm^{-1} with stick spectra of seven isomers of $H^{+}C_{14}H_{10}$. (a) and (b) are experimental spectra taken from Figure S5. (a) Inverted difference spectrum measured after maintenance of the matrix in darkness for 16 h. (b) Difference spectra after secondary irradiation at 544 nm for 20 min. Lines in group X are indicated with purple labels and lines. Spectral regions subjected to interference from the intense absorption of $C_{14}H_{10}$ are shaded gray. Baselines are shifted for clarity. The stick spectra (c), (d), and (f)−(j) are based on scaled harmonic vibrational wavenumbers and IR intensities calculated with the B3LYP/6-311++G(d,p) method. The anharmonic vibrational stick spectrum of 1-$H^{+}C_{14}H_{10}$ is shown in trace (e), with combination and overtone bands in light color.

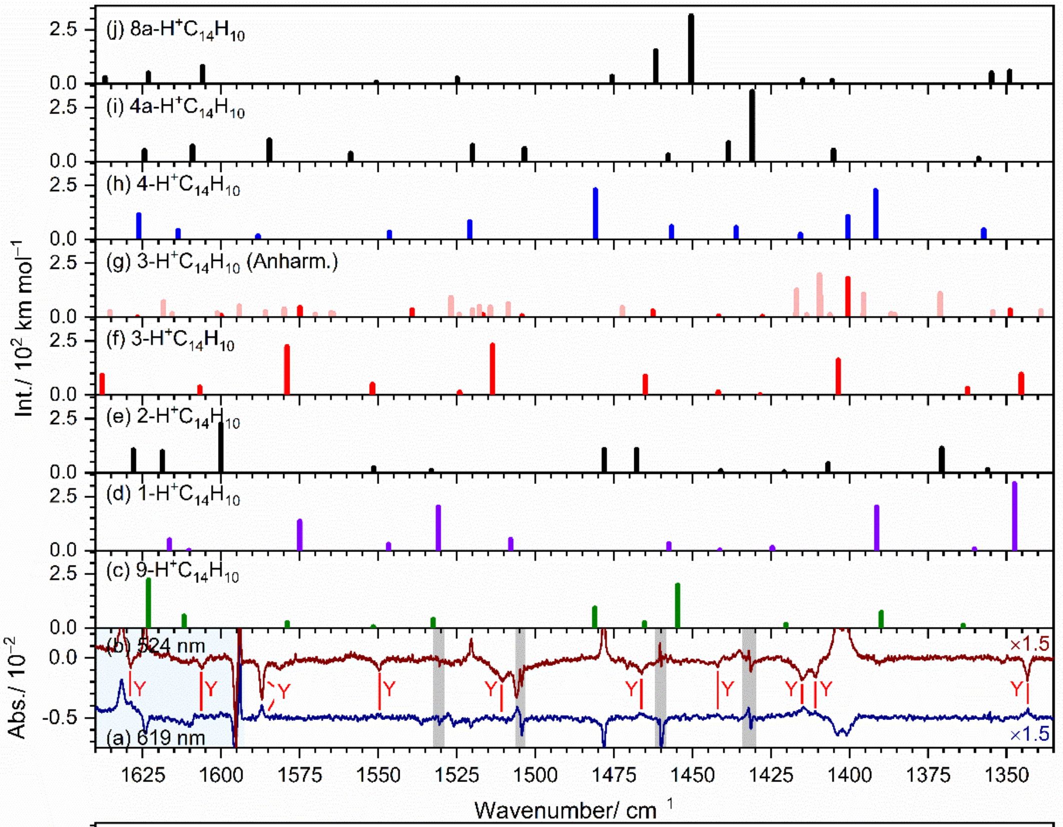
(j) 8a-H+C14H10
(i) 4a-H+C14H10
(h) 4-H+C14H10
(g) 3-H+C14H10 (Anharm.)
(f) 3-H+C14H10
(e) 2-H+C14H10
(d) 1-H+C14H10
(c) 9-H+C14H10
(b) 524 nm
(a) 619 nm
Int./ 10^2 km mol^-1
Abs./ 10^-2
×1.5
Wavenumber/ cm^-1

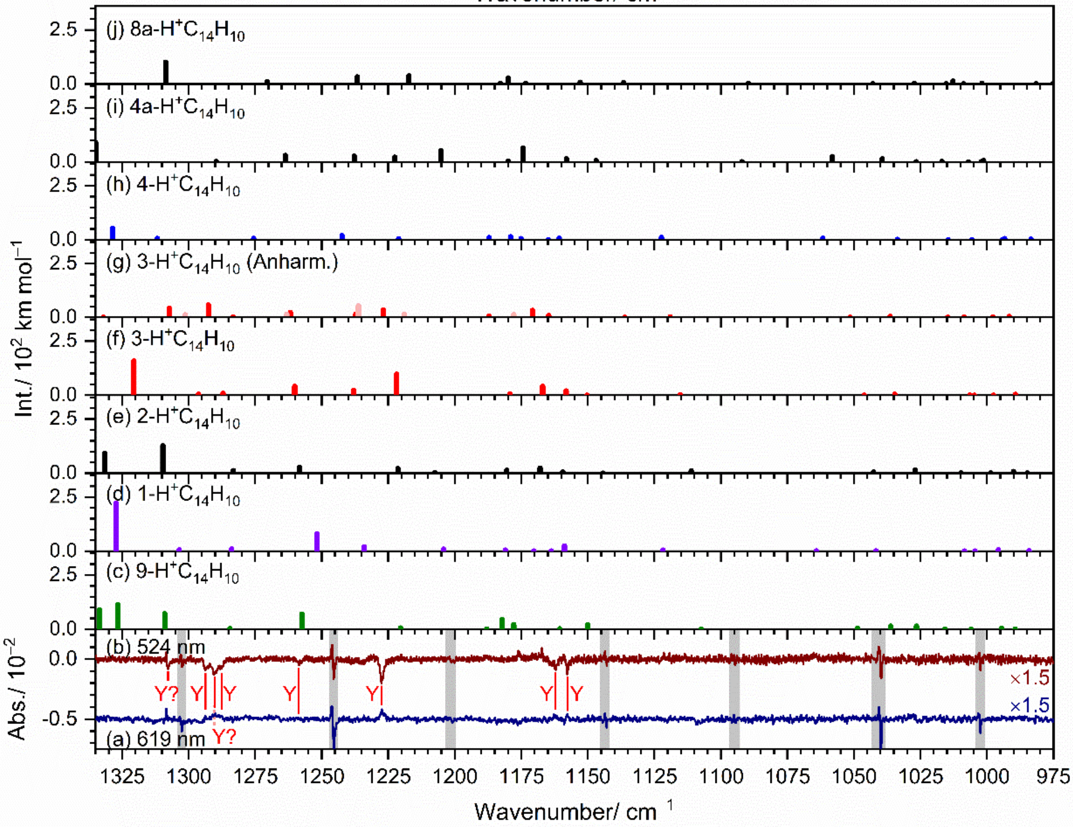
(j) 8a-H+C14H10
(i) 4a-H+C14H10
(h) 4-H+C14H10
(g) 3-H+C14H10 (Anharm.)
(f) 3-H+C14H10
(e) 2-H+C14H10
(d) 1-H+C14H10
(c) 9-H+C14H10
(b) 524 nm
(a) 619 nm
Y?
Int./ 10^2 km mol^-1
Abs./ 10^-2
×1.5
Wavenumber/ cm^-1

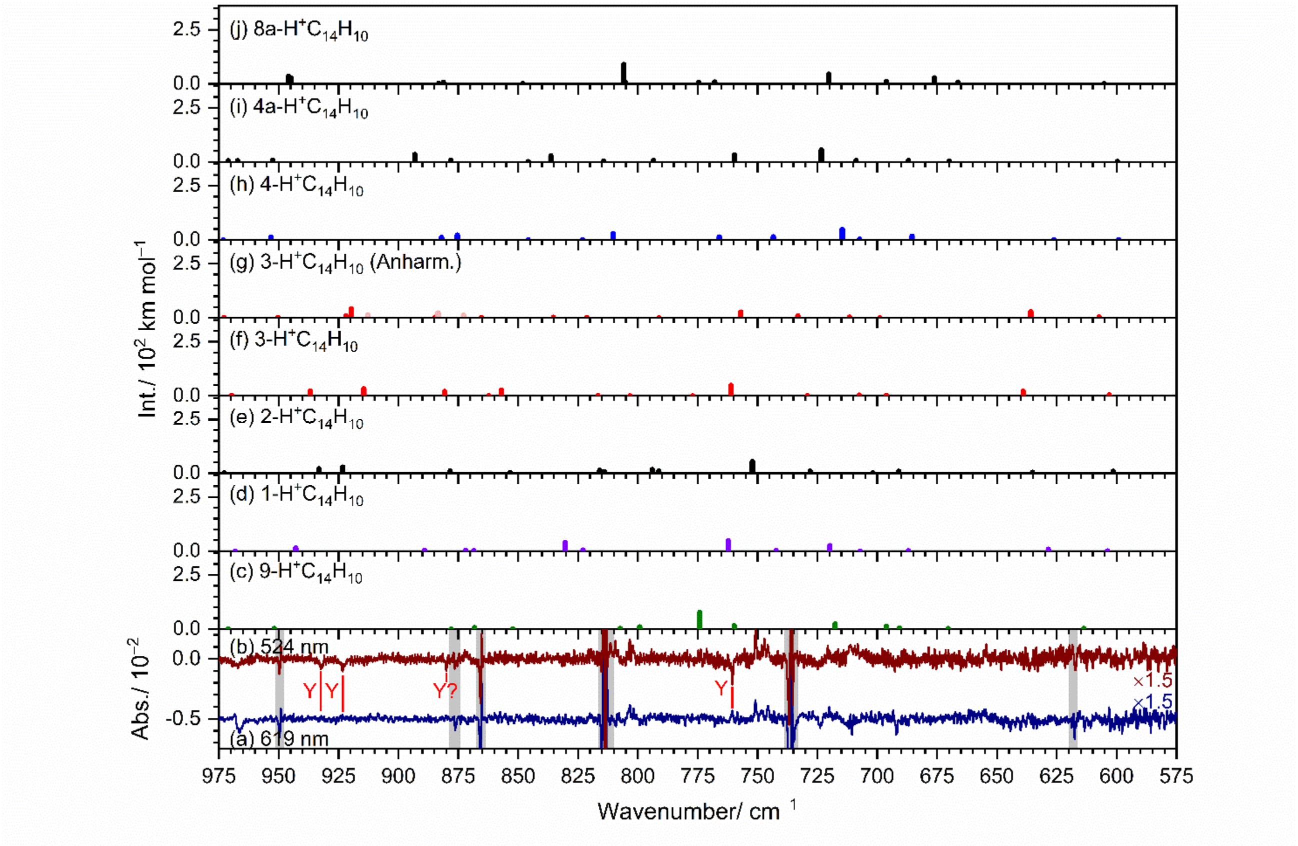

Figure S9. Comparison of lines in group Y in region 575−1640 cm^{-1} with stick spectra of seven isomers of $H^+C_{14}H_{10}$. Difference spectra after secondary irradiation at 619 nm (a) and 524 nm (b) are experimental spectra taken from Figure S5. Lines in group Y are indicated with red labels and lines. Spectral regions subjected to interference from the intense absorption of $C_{14}H_{10}$ are shaded gray. Baselines are shifted for clarity. The stick spectra (c)−(f) and (h)−(j) are based on scaled harmonic vibrational wavenumbers and IR intensities calculated with the B3LYP/6-311++G(d,p) method. The anharmonic vibrational stick spectrum of 3-$H^+C_{14}H_{10}$ is shown in trace (g), with combination and overtone bands in light color.

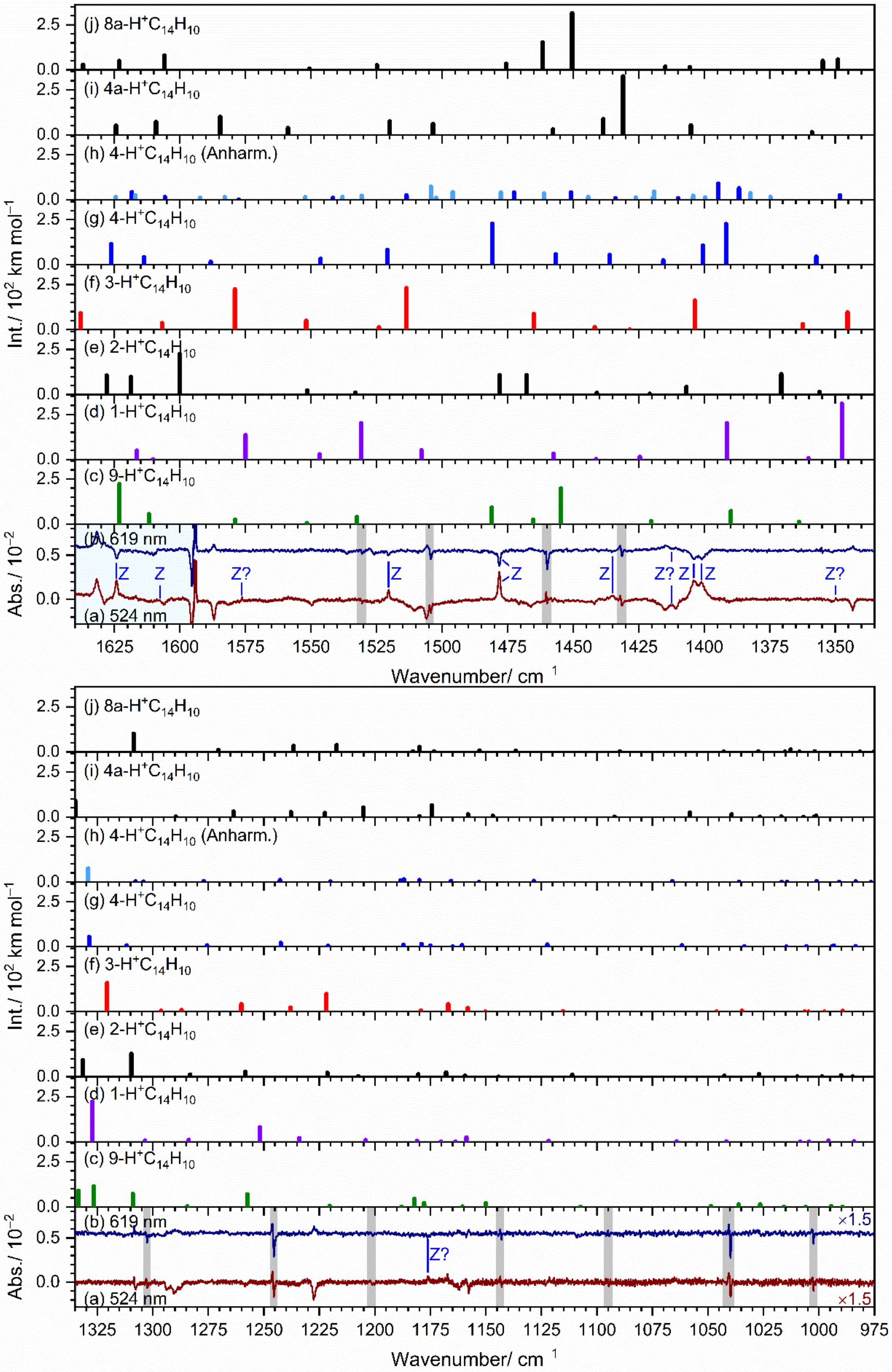
(j) 8a-H+C14H10
(i) 4a-H+C14H10
(h) 4-H+C14H10 (Anharm.)
(g) 4-H+C14H10
(f) 3-H+C14H10
(e) 2-H+C14H10
(d) 1-H+C14H10
(c) 9-H+C14H10
(b) 619 nm
(a) 524 nm
Int./ 10^2 km mol^-1
Abs./ 10^-2
Wavenumber/ cm^-1
Z
Z?
×1.5

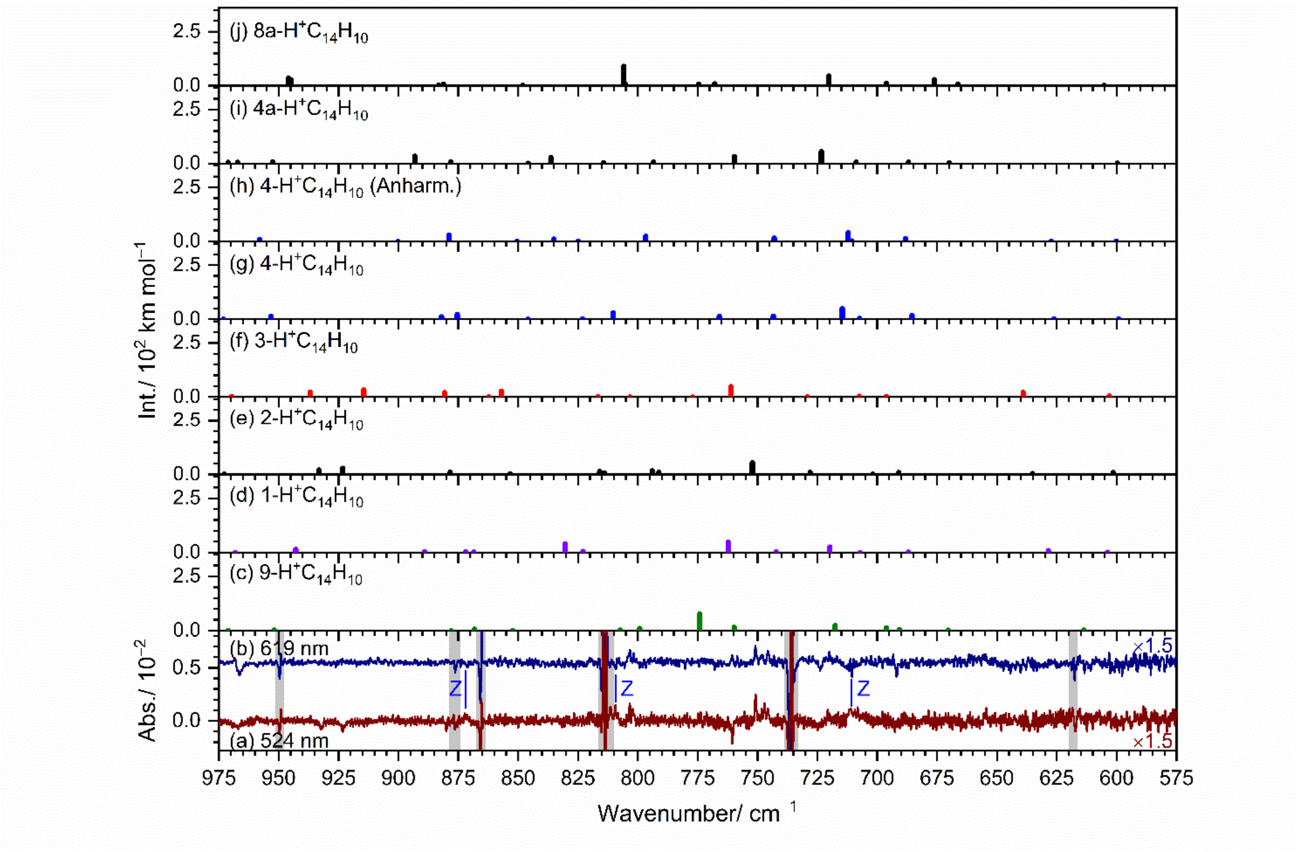

Figure S10. Comparison of lines in group Z in region 575−1640 cm^{-1} with stick spectra of seven isomers of $H^+C_{14}H_{10}$. The stick spectra are based on scaled harmonic vibrational wavenumbers and IR intensities calculated with the B3LYP/6-311++G(d,p) method. Difference spectra after secondary irradiation at 524 nm (a) and 619 nm (b) are experimental spectra taken from Figure S5. Lines in group Z are indicated with blue labels and lines. Spectral regions subjected to interference from the intense absorption of $C_{14}H_{10}$ are shaded gray. Baselines are shifted for clarity. The stick spectra (c)−(g), (i), and (j) are based on scaled harmonic vibrational wavenumbers and IR intensities calculated with the B3LYP/6-311++G(d,p) method. The anharmonic vibrational stick spectrum of 4-$H^+C_{14}H_{10}$ is shown in trace (h), with combination and overtone bands in light color.

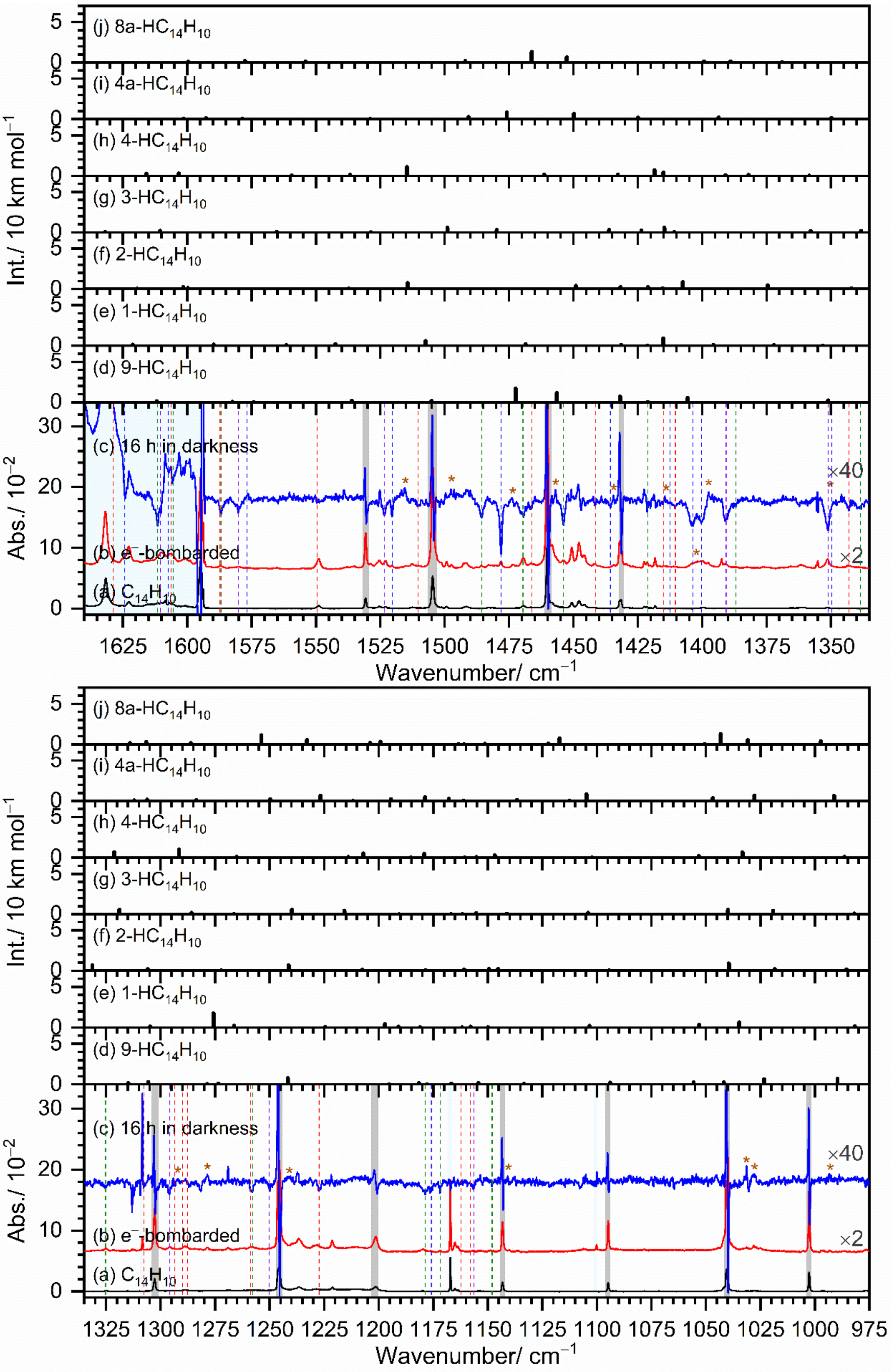
(j) 8a-$HC_{14}H_{10}$
(i) 4a-$HC_{14}H_{10}$
(h) 4-$HC_{14}H_{10}$
(g) 3-$HC_{14}H_{10}$
(f) 2-$HC_{14}H_{10}$
(e) 1-$HC_{14}H_{10}$
(d) 9-$HC_{14}H_{10}$
(c) 16 h in darkness
(b) e^--bombarded
(a) $C_{14}H_{10}$
×40
×2
Int./ 10 km mol^{-1}
Abs./ 10^{-2}
Wavenumber/ cm^{-1}

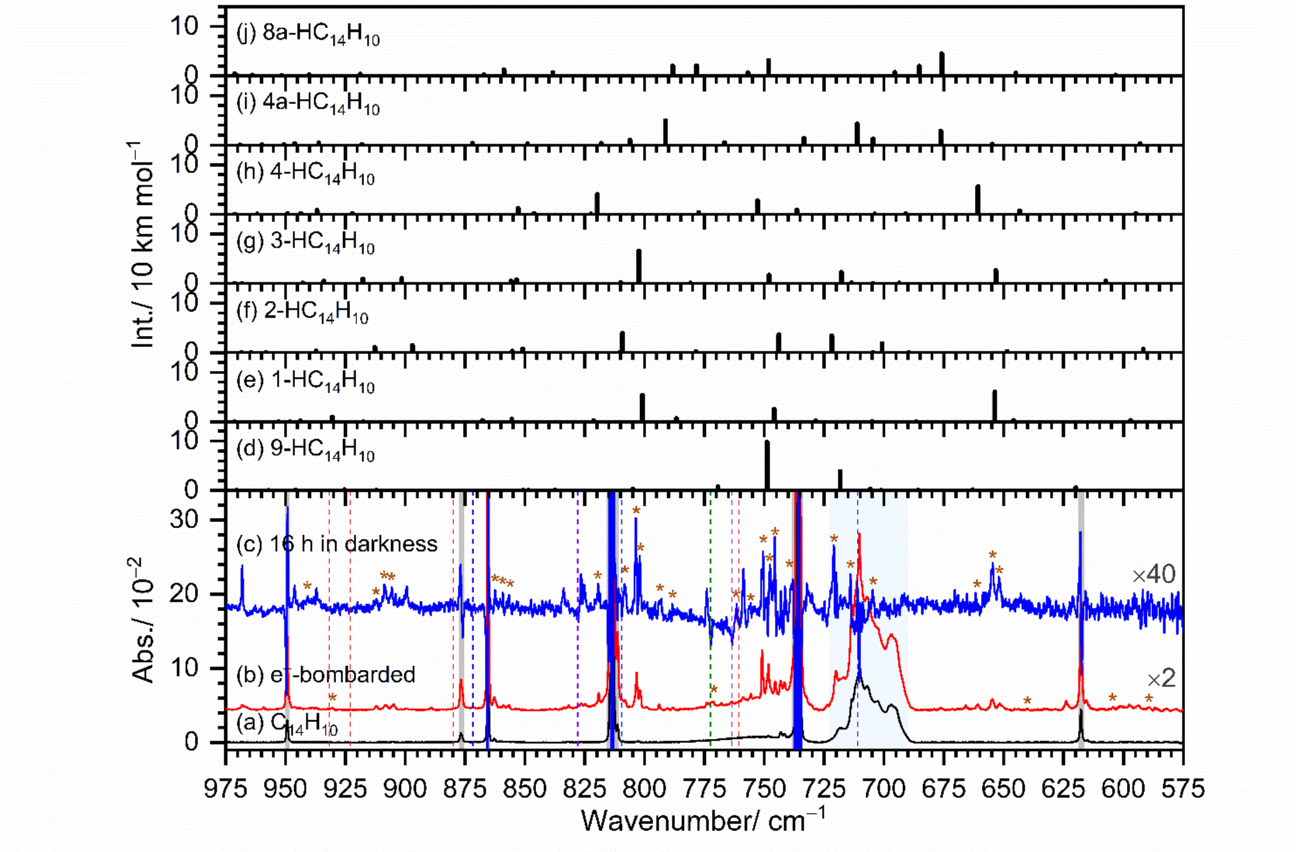

Figure S11. Comparison of lines of $H^+C_{14}H_{10}$ and $HC_{14}H_{10}$ in region 575−1640 cm^{-1} with stick spectra of isomers of $HC_{14}H_{10}$. Absorption spectra of $C_{14}H_{10}$ (a), those after electron bombardment (b), and after maintenance in darkness for 16 h (c) are shown with baseline shifted for clarity. Lines of $H^+C_{14}H_{10}$ are indicated with color-coded dashed lines; the color coding is the same as in other figures. Features of $HC_{14}H_{10}$ are indicated with * marks. Spectral regions subjected to interference from the intense absorption of $C_{14}H_{10}$ are shaded gray. The stick spectra (d)−(j) are based on scaled harmonic vibrational wavenumbers and IR intensities calculated with the B3LYP/6-311++G(d,p) method.

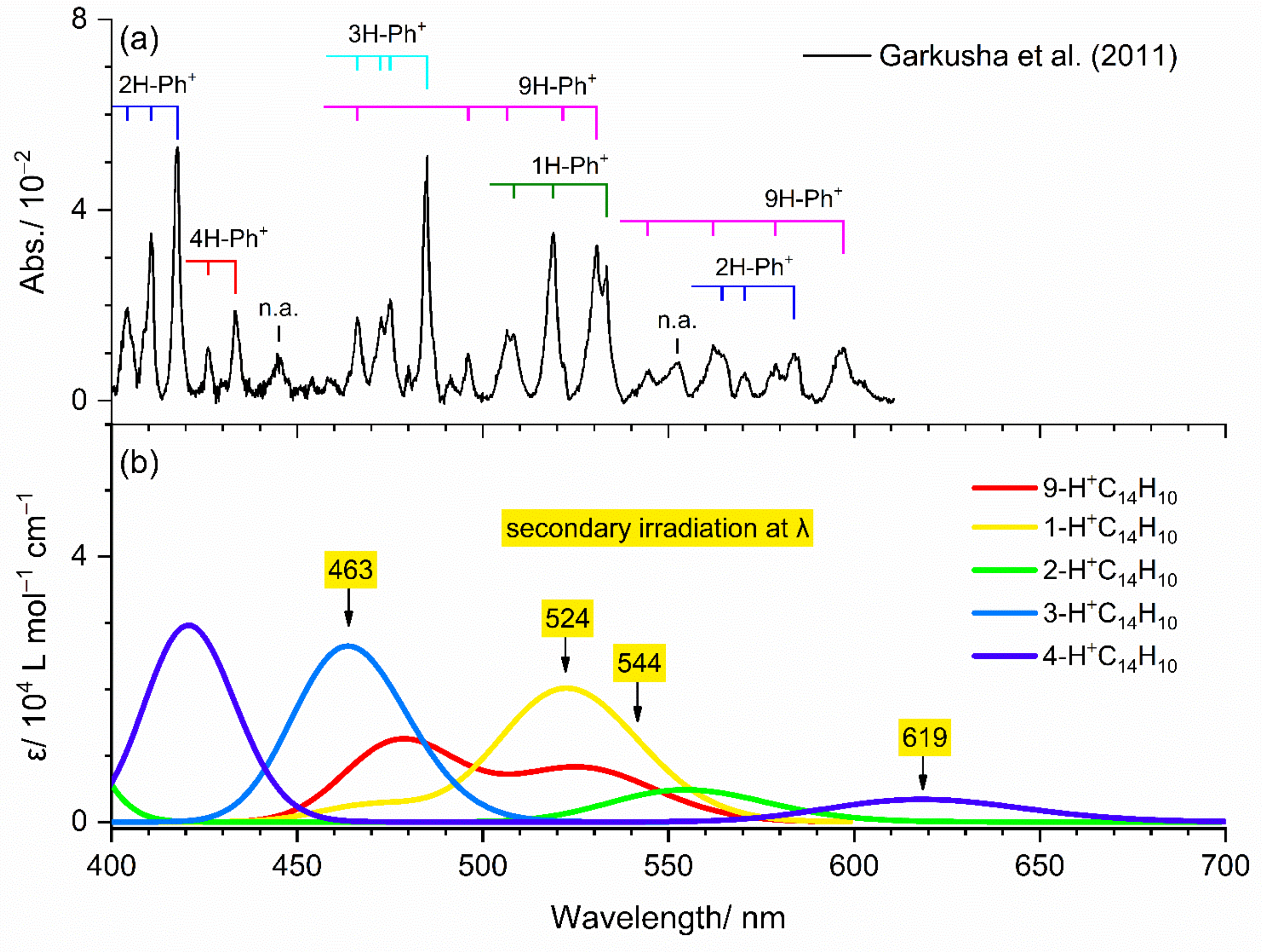

Figure S12. Experimental UV spectra and calculated vertical electronic excitation spectra of 9-, 1-, 2-, 3-, and 4-$H^+C_{14}H_{10}$. (a) Experimental absorption spectrum taken from Garkusha et al. [4]. Reproduced with permission from [4]. Copyright [2011] Astrophys. J. (b) The spectra are fitted with a Gaussian function with a full-width-at-half-maximum of 0.1 eV. The numbers in yellow squares are the selected wavelengths (in nm) for secondary irradiation. The experimental progression starting at 596.6 nm might have to be assigned to 4-$H^+C_{14}H_{10}$ instead of 9-$H^+C_{14}H_{10}$, according to quantum-chemical calculations and our experimental observations.

References

(1) Hudgins, D. M.; Sandford, S. A. Infrared Spectroscopy of Matrix Isolated Polycyclic Aromatic Hydrocarbons. 1. PAHs Containing Two to Four Rings. *J. Phys. Chem. A* **1998**, *102*, 329–343.

(2) Langhoff, S. R. Theoretical Infrared Spectra for Polycyclic Aromatic Hydrocarbon Neutrals, Cations, and Anions. *J. Phys. Chem.* **1996**, *100*, 2819–2841.

(3) Cané, E.; Miani, A.; Palmieri, P.; Tarroni, R.; Trombetti, A. The gas-phase infrared spectra of phenanthrene-h_{10} and phenanthrene-d_{10}. *Spectrochim. Acta A* **1997**, *53*, 1839–1851.

(4) Garkusha, I.; Fulara, J.; Nagy, A.; Maier, J. P. Electronic Absorption Spectra of Protonated Anthracenes and Phenanthrenes, and Their Neutrals in Neon Matrices. *Astrophys. J.* **2011**, *728*, 131.
